# Brain Region-Specific Gene Signatures Revealed by Distinct Astrocyte Subpopulations Unveil Links to Glioma and Neurodegenerative Diseases

**DOI:** 10.1523/ENEURO.0288-18.2019

**Published:** 2019-04-02

**Authors:** Raquel Cuevas-Diaz Duran, Chih-Yen Wang, Hui Zheng, Benjamin Deneen, Jia Qian Wu

**Affiliations:** 1 The Vivian L. Smith Department of Neurosurgery, McGovern Medical School, University of Texas Health Science Center at Houston, Houston, Texas 77030; 2 Center for Stem Cell and Regenerative Medicine, UT Brown Foundation Institute of Molecular Medicine, Houston, Texas 77030; 3 Tecnologico de Monterrey, Escuela de Medicina y Ciencias de la Salud, Monterrey NL 64710, Mexico; 4 Department of Life Sciences, National Cheng Kung University, Tainan City 70101, Taiwan; 5 Huffington Center on Aging; 6 Medical Scientist Training Program; 7 Department of Molecular and Human Genetics; 8 Center for Cell and Gene Therapy; 9 Department of Neuroscience; 10 Neurological Research Institute at Texas’ Children’s Hospital; 11 Program in Developmental Biology, Baylor College of Medicine, Houston, Texas 77030

**Keywords:** astrocyte subpopulations, gene set variation analysis, gene signatures, genomic alterations, glioma, neurodegenerative disorders

## Abstract

Currently, there are no effective treatments for glioma or for neurodegenerative diseases because of, in part, our limited understanding of the pathophysiology and cellular heterogeneity of these diseases. Mounting evidence suggests that astrocytes play an active role in the pathogenesis of these diseases by contributing to a diverse range of pathophysiological states. In a previous study, five molecularly distinct astrocyte subpopulations from three different brain regions were identified. To further delineate the underlying diversity of these populations, we obtained mouse brain region-specific gene signatures for both protein-coding and long non-coding RNA and found that these astrocyte subpopulations are endowed with unique molecular signatures across diverse brain regions. Additional gene set and single-sample enrichment analyses revealed that gene signatures of different subpopulations are differentially correlated with glioma tumors that harbor distinct genomic alterations. To the best of our knowledge, this is the first study that links transcriptional profiles of astrocyte subpopulations with glioma genomic mutations. Furthermore, our results demonstrated that subpopulations of astrocytes in select brain regions are associated with specific neurodegenerative diseases. Overall, the present study provides a new perspective for understanding the pathophysiology of glioma and neurodegenerative diseases and highlights the potential contributions of diverse astrocyte populations to normal, malignant, and degenerative brain functions.

## Significance Statement

Mounting evidence suggests that astrocytes play an active role in disease pathogenesis by contributing to a diverse range of pathophysiological states. The present study identifies new layers of astrocyte diversity and new correlations between distinct astrocyte subpopulations and disease states. Understanding the heterogeneity of astrocytes in different physiologic conditions and diseases will provide new avenues for improved diagnostics and therapeutics targeting specific astrocyte subpopulations.

## Introduction

Astrocytes make up ∼40% of all cells in the human brain (Herculano-Houzel, 2014; Zhang et al., 2016) and play essential roles in brain function by controlling extracellular neurotransmitter and potassium ion levels, regulating the blood–brain barrier, and promoting synapse formation and function (Barres, 2008). Previously, astrocytes were thought to be a homogeneous population of cells that tile the CNS and support neuronal survival. However, recent genome-wide gene expression studies have shown that astrocytes are highly heterogeneous (Cahoy et al., 2008; Doyle et al., 2008; Yeh et al., 2009). A recent study demonstrated astrocyte heterogeneity in the brain by comparing gene expression profiles of FACS-sorted Aldh1l1-GFP^+^ subpopulations and identified five astrocyte subpopulations (A–E) across three brain regions (olfactory bulb, cortex, and brainstem). Moreover, these astrocyte subpopulations exhibited functional differences related to proliferation, migration, and synapse formation (John Lin et al., 2017).

Functional heterogeneity of astrocytes has been described under both normal physiologic conditions and diverse disease states (Denis-Donini et al., 1984; Hill et al., 1996; Grass et al., 2004; White et al., 2010; Morel et al., 2017). During disease progression, astrocyte populations respond to pathologic conditions through a transformation called *reactive astrogliosis*. Morphologic and gene expression differences have been observed in reactive astrocytes from different brain regions during the progression of neurodegenerative diseases (Hill et al., 1996; Liddelow et al., 2017). Nervous system malignancies also have astroglial origins. Gliomas, the most common primary malignancies in the CNS, are a heterogeneous group of tumors characterized by their resemblance to glia. A parallel between brain development and glioma tumorigenesis is apparent in that certain astrocyte subpopulations are analogous to those that populate human glioma (John Lin et al., 2017). Despite the discovery of these links between diverse astrocyte populations and disease states, how distinct subpopulations of astrocytes contribute to certain neurologic disease remains poorly defined. For example, given the plethora of genomic data available for human glioma, whether key genetic mutations associated with glioma are also associated with specific astrocyte/glioma subpopulation remains unknown. Thus, one of the goals of this study is to identify new correlations between diverse astrocyte subpopulations and distinct disease states.

In addition to correlations with disease states, another goal of this study is to unearth additional layers of astrocyte heterogeneity. A novel study identified five distinct subpopulations of astrocytes across three brain regions (John Lin et al., 2017). However, astrocyte subpopulations between different brain regions were not compared, and the expression of long non-coding RNAs (lncRNAs), a type of regulatory RNA that has been shown to play important roles in CNS development, plasticity, and disease (Hung et al., 2011; Guttman and Rinn, 2012; Batista and Chang, 2013; Zhang et al., 2014; Dong et al., 2016) was not investigated. Thus, the other goal of this study is to further delineate the underlying molecular diversity of astrocytes from existing datasets.

Toward the overarching goal of decoding the nature of astrocyte diversity, we used advanced comparative bioinformatics to obtain region-specific (e.g., cortex-specific) and regional subpopulation-enriched (e.g., cortex subpopulation A-enriched) astrocyte gene signatures. In addition to these 15 astrocyte regional subpopulations (5 subpopulations in 3 brain regions), we also collected gene expression datasets for other astrocyte subpopulations from different developmental stages and injury models (Rusnakova et al., 2013; Zeisel et al., 2015; Gokce et al., 2016; Noristani et al., 2016; Hara et al., 2017; Wu et al., 2017). Our analysis included a total of 42 astrocyte gene signatures. We implemented an unsupervised single-sample enrichment method to correlate astrocyte subpopulation gene signatures with The Cancer Genome Atlas (TCGA) lower-grade glioma (LGG), and glioblastoma (GBM) samples including clinical sample features and key mutations. The results show that specific astrocyte subpopulation gene signatures are more highly correlated with certain glioma subtypes and genomic variants. For example, astrocyte subpopulations B and C in all brain regions are significantly correlated with amplification of the gene encoding EGFR (epidermal growth factor receptor). Additionally, astrocyte subpopulations D and E were among the gene signatures highly correlated with LGG samples bearing both mutation in *IDH* gene and 1p/19q codeletion. Furthermore, correlations between astrocyte subpopulations and neurodegenerative diseases identified a collection of genes that we validated in Alzheimer's disease (AD) mouse models. Together, the present study identifies new layers of astrocyte diversity, while making critical new connections between these populations and neurologic disease including glioma and neurodegeneration. Thus, understanding astrocyte subpopulations has a broad utility that spans normal, malignant, and degenerative brain functions, and will open new avenues for the development of improved diagnostics and therapeutics to target specific astrocyte subpopulations.

## Materials and Methods

### Astrocyte subpopulation- and brain region-specific gene signatures

RNA-seq datasets for five astrocyte subpopulations (A, B, C, D, E) from three brain regions (olfactory bulb, brainstem, and cortex) were downloaded from the data repository for a previous publication (GSE72826; John Lin et al., 2017). Data were obtained from both male and female mice. For each brain region, non-astrocyte samples (Aldh1l1-GFP^−^ cells) were also downloaded. Briefly, reads were mapped to the mm10 mouse reference genome downloaded from GENCODE (https://www.gencodegenes.org/) using TopHat v2.1.0 (Trapnell et al., 2009). Mapped reads were assembled using Cufflinks v2.2.1 (Trapnell et al., 2012) and Fragments per kilobase of transcript per million mapped reads (FPKM) values were obtained for both protein-coding and lncRNA genes using a published pipeline (Dong et al., 2015; Cuevas Diaz Duran et al., 2016; Wu, 2016; Duran et al., 2017). Our annotation file included 21,948 protein-coding genes (55,252 transcripts) and 29,232 lncRNA genes (49,189 transcripts). Any value of FPKM <0.1 was set to 0.1 to avoid ratio inflation (Quackenbush, 2002). Additionally, read counts for all annotated genes and transcripts were calculated using HTSeq-count (Anders et al., 2015). FPKM and normalized read count matrices are included in Extended data Figure 2-1.

**Figure 1.**
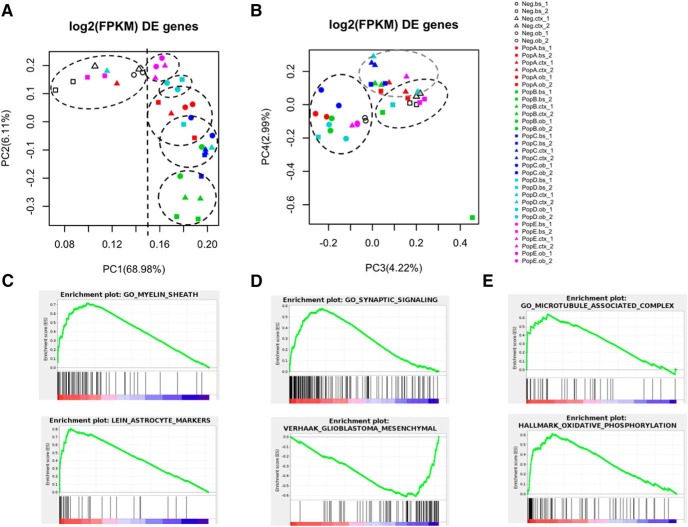
PCA performed using log2-transformed FPKM of 4092 genes DE between regions. ***A***, Scores for PC1 and PC2 are depicted. PC1 separates samples into astrocyte subpopulations and non-astrocyte populations. Circular and elliptical figures enclose samples according to subpopulation. ***B***, The plot of PC3 and PC4 separates samples into brain regions, as indicated by the elliptical figures. ***C***–***E***, Differentially expressed genes were ranked using the scores of PC1, PC2, and PC3, respectively, and the top enriched gene sets were obtained through GSEA.

**Figure 2.**
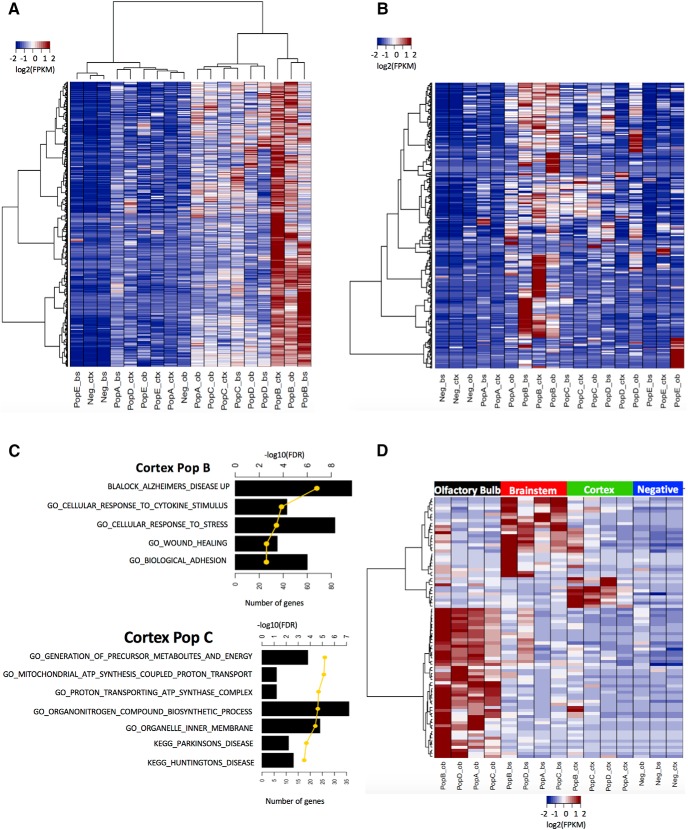
Gene signatures defined by astrocyte subpopulations and brain regions. ***A***, Heatmap showing the log2-transformed FPKM values of cortex subpopulation B-enriched genes (protein-coding and lncRNAs) compared with other subpopulations. DE genes were obtained by performing pairwise comparisons of astrocyte subpopulations in each brain region to their corresponding non-astrocyte samples. Selection criteria: FPKM > 1, fold-change > 2, and FDR < 10%. ***B***, Heatmap illustrating upregulated DE lncRNA genes from astrocyte regional subpopulation gene signatures. ***C***, The top five significantly enriched gene sets obtained using the gene signatures of cortex subpopulation B (top) and cortex subpopulation C (bottom). Bar plots indicate the number of genes found in the enriched gene set. The yellow line illustrates the gene set enrichment using -log10-transformed FDR. ***D***, Heatmap depicting the region-specific astrocyte gene signatures. DE genes were obtained by comparing all samples from the same brain region to the remaining regions (FPKM > 1, fold-change > 4, and *p* < 0.05) and to their corresponding non-astrocyte samples (FPKM > 1, fold-change > 2, and *p* < 0.05). Colors of all heatmaps represent log2 transformed FPKM values. See also Extended data Figures 2-1, 2-2, 2-3, 2-4.

10.1523/ENEURO.0288-18.2019.f2-1 Figure 2-1 Gene expression as FPKM and normalized read count matrices across astrocyte subpopulations. Download Figure 2-1, XLSX file.

To obtain regional subpopulation-enriched gene signatures (e.g., for cortex subpopulation A), we compared astrocyte subpopulations to non-astrocytes within each region in a pairwise mode. Comparisons were performed using DESeq2 (Love et al., 2014) with normalized counts. A binary results matrix was created with three rows (regions) and five columns (subpopulations). Each cell in the matrix represented a comparison of an astrocyte subpopulation from a specific region against the non-astrocyte sample in the same region. These were referred to as *regional subpopulation*-enriched gene signatures. Genes were considered significant if they were expressed (FPKM >1) in at least one of the replicates with an expression fold-change >2 and FDR <0.1%. The log-transformed fold-changes and *q* values of differentially expressed protein-coding and lncRNA genes from different astrocyte subpopulations and regions were compared using a *t* test (*p* < 0.05). (The details of all statistical analyses are listed in Table 1).

**Table 1.** List of figures for each experiment indicating data structure, statistical tests applied, and significance levels

**Figure**	**Data structure**	**Type of test**	**Power**	**Notes**
Fig. 1*C*–*E*	GSEA	Kolmogorov–Smirnov	FDR < 0.01	|NES| > 2
Fig. 2*A*,*B*	Differential expression analysis	DESeq2 generalized linear model (GLM)	FDR < 0.1	FPKM > 1, normalized count fold-change > 2
Fig. 2*C*	Gene set enrichment	Hypergeometric	FDR < 0.05	Gene no. > 5
Fig. 2*D*	Differential expression analysis	DESeq2 GLM	*p* < 0.05	FPKM > 1, normalized counts, fold-change >4 (comparedwith astrocyte subpopulations from other regions),fold-change > 2 (compared with non-astrocyte sample).
Fig. 2-2	Normal distribution	Unpaired Student’s *t* test	*p* < 0.05	
Figs. 3*C*, 4*B*	Correlations	ANOVA, Wilcoxon rank sum tests	*p* < 0.05 (FDR < 0.25)	
Fig. 5	Kaplan–Meier survival plots	Log rank test Fisher’s test	*p* < 0.05	
Fig. 6*A–C*, Fig. 6-2*B*–*D*	Immunostained images	Two-tailed unpaired Student’s *t* test	*p* < 0.05, *p* < 0.01, *p* < 0.001	

10.1523/ENEURO.0288-18.2019.f2-2 Figure 2-2 Comparison of log-transformed fold-changes and *q* values between DE lncRNA and protein-coding genes from different astrocyte subpopulation and region gene signatures. Blue circles represent DE lncRNAs and red circles represent DE protein-coding genes. Using an unpaired two-tailed *t* test we observed that the fold-changes of DE lncRNAs (blue) are statistically higher (*p* < 0.05) than those of protein-coding genes (red). Download Figure 2-2, TIF file.

To identify region-specific gene signatures, we compared all astrocyte samples from one region to astrocyte samples from the remaining two regions, yielding a total of three comparisons. Comparisons were performed using DESeq2 (Love et al., 2014) with normalized counts. Additionally, astrocyte samples from the same region were compared with their corresponding non-astrocyte samples. A gene was differentially expressed (DE) with FPKM > 1 in at least one of the replicates in each comparison, with an expression fold-change > 4 at *p* < 0.05, and a fold-change against non-astrocyte samples > 2 at *p* < 0.05.

## Principal components analysis

Principal components analysis (PCA) is an unsupervised, exploratory, and multivariate statistical technique used to reduce the dimensionality of complex datasets while retaining most of the variation (Jolliffe, 2002). PCA identifies vectors, or principal components (PCs), for which the variation in the data are maximal. Because each PC is a linear combination of the original variables, it is possible to identify a biological interpretation of each component that explains the differences between the samples. When using PCA in genome-wide expression studies, the dataset generally consists of a gene expression matrix with genes as rows and samples as columns.

PCA was implemented by first generating a gene expression matrix of log2-transformed FPKM values of DE genes for all the samples and their replicates, with regions as rows and samples as columns, to yield a 4092 × 36 matrix. The “prcomp” R function (R Core Team, 2015), which computes the singular value decomposition of the gene expression matrix, was then implemented. Samples were considered as variables and PCs that captured most of the variability in the samples were obtained.

To gain insight into the biological significance of the identified PCs and their underlying regulatory factors, gene set enrichment analysis (GSEA; Subramanian et al., 2005) was performed. DE genes were ranked by the scores assigned according to each PC. Using the MsigDB gene set database (Liberzon et al., 2011), the top enriched gene sets with FDR < 0.01 and absolute normalized enrichment score (NES) > 2 were deemed significant.

### Collection of astrocyte gene signatures

In addition to the 15 astrocyte regional subpopulations (5 subpopulations in 3 brain regions) previously described, we collected 25 gene expression datasets for other astrocyte subpopulations from diverse studies in which astrocytes were purified using single-cell microfluidics (Zeisel et al., 2015; Gokce et al., 2016; Wu et al., 2017), FACS-sorting (Rusnakova et al., 2013; Noristani et al., 2016), or laser microdissection (Hara et al., 2017). These diverse astrocyte subpopulations consisted of purified cells from brain (amygdala, striatum, hippocampus, and cortex), different developmental stages, and from spinal cord injury models. We used astrocyte gene signatures including both protein-coding and lncRNA genes except those gene signatures obtained from literature where lncRNA information was not reported. A brief description of each of these gene profiles is included in Extended data Figure 3-1. To determine the similarities between astrocyte gene signatures, we calculated the Jaccard index. Indexes >0.7 were considered significant.

### Gene set variation analysis

Glioma RNA-Seq data (counts) were downloaded from the Recount2 database (Frazee et al., 2011). Clinical data from TCGA GBM dataset was collected from the Broad Institute GDAC Firehose website (v2016_01_28) and from the GlioVis website (Bowman et al., 2017). For TCGA LGG dataset, the genomic data from the supplemental materials of the PanGlioma TCGA paper were collected (Ceccarelli et al., 2016). Our analysis included sample features comprising gene expression, histology, subtype, somatic mutations, copy-number variations, 1p/19q codeletions, and other clinical features.

To compare astrocyte subpopulations and glioma samples, we adopted the gene set variation analysis (GSVA) approach (Hänzelmann et al., 2013). GSVA is an unsupervised, nonparametric method used to evaluate the degree to which genes in a gene signature are coordinately up- or downregulated within a particular biological sample. The GSVA R package was used to calculate an enrichment score for each astrocyte gene signature across individual glioma samples and frequently encountered somatic mutations to determine whether an astrocyte subpopulation is associated with those glioma subtypes or genomic variations. The genomic features used for GBM samples included transcriptional subtype; mutations in genes encoding TP53, EGFR, PTEN, NF1, PIK3R1, RB1, ATRX, IDH1, APOB, or PDGFRA; amplifications of genes encoding EGFR, PDGFRA, or CDK4; and deletions of genes encoding MTAP, PTEN, QKI, or RB1. Similarly, for LGG, the genomic features queried included tumor grade, histologic subtype, mutation in the *IDH* gene, codeletion of 1p/19q loci, mutations in genes encoding TP53, EGFR, PTEN, ATRX, IDH1, IDH2, CIC, NOTCH1, FUBP1, PIK3CA, NF1, PIK3R1, SMARCA4, ARID1A, TCF12, ZBTB20, PTPN11, PLCG1, or ZCCHC12; deletions of genes encoding PTEN, NF1, CDKN2C, and CDKN2A; and amplifications of genes encoding PIK3CA, PIK3C2B, PDGFRA, MDM4, MDM2, EGFR, or CDK4.

To evaluate whether the differences among groups of samples with different genomic features (such as subtype, mutations, and copy-number variations) could be explained using GSVA enrichment scores, ANOVA and Wilcoxon rank sum tests were conducted. Each genomic feature was correlated to all of the gene signatures by comparing enrichment scores between groups of samples formed according to the different levels of the genomic feature. A correlation matrix of transformed *p* values was obtained for GBM and LGG samples. Correlations were deemed significant with *p* < 0.05 (FDR < 0.25 using the Benjamini and Hochberg adjustment for multiple comparisons). GSVA enrichment score matrices and correlation matrices of transformed *p* values are included in Extended data Figure 3-6.

### Survival analysis

Correlations between astrocyte gene signatures and patient survival were calculated to conduct Kaplan–Meier survival analyses. Clinical data were parsed to obtain patients’ age, vital status (dead/alive), and months to death or months from most recent follow-up depending on the patients’ vital status. Patient samples were filtered to use only those with tumor purity >70%. Classical GBM samples with EGFR amplification and LGG samples with astrocytoma histology were used for survival analyses. For each astrocyte gene signature, samples were categorized into two groups: (1) High (enrichment scores >0) and (2) Low (enrichment scores <0). Kaplan–Meier survival plots were generated for each astrocyte gene signature using the “survival” (Therneau, 2015) and “survminer” (Kassambara and Kosinski, 2017) R libraries. The survival curves of samples with High and Low enrichment scores were compared using log rank tests. To determine the combined survival effect of score enrichment and age, we applied multivariate Kaplan–Meier survival analysis. For example, to estimate the combined effect of an astrocyte gene signature and age, the samples were first divided according to whether they had High or Low enrichment scores. Then within each group, samples were further stratified according to the age at which the tumor was diagnosed. Samples were grouped by age as either younger or older than 60 years (for GBM samples), or as 45 years (for LGG samples). Age thresholds were obtained by creating a histogram of the “age at diagnosis” and selecting the age at which the distribution is divided into two balanced subdistributions representing the younger and the older groups. The *p* value obtained from the log rank test was used to indicate the statistical significance of correlations in survival between groups. Furthermore, to determine the association of *IDH* mutations with the survival of samples correlated with astrocyte gene signatures we constructed contingency tables and performed Fisher’s tests. Associations with *p* values < 0.05 were considered significant.

### GSEA

A comprehensive collection of gene sets was downloaded from the Molecular Signatures database (MsigDB; Liberzon et al., 2011). Additional gene sets were obtained from neurodegenerative disease studies in which gene expression was assessed (Extended data Fig. 2-4). A hypergeometric statistical test (phyper R function) was used to determine gene set enrichment using the DE gene list for each astrocyte subpopulation, region, and regional subpopulation. Gene sets were considered enriched with FDR < 0.05 and gene number > 5.

### Immunofluorescence

Brain tissues from 5xFamilial Alzheimer’s disease (5xFAD; Polito et al., 2014) and APP^NLGF^ (NLGF; Saito et al., 2014; gifts from Dr. Zheng Hui, Huffington Center on Aging, Baylor College of Medicine) mice were fixed in 4% paraformaldehyde and sectioned at 20 μm thickness. The brain tissues were permeabilized with 0.3% Triton™ X-100 (ThermoFisher Scientific) in PBS and blocked with 2.5% horse serum (Vector Laboratories) for 1 h at room temperature. The sections were then incubated in PBS with 0.1% Triton X-100 containing chicken anti-GFAP (1:500; Abcam) combined with either rabbit anti-Adcy7 (1:100; Bioss), mouse anti-Serping1 (1:50; Santa Cruz Biotechnology), or rabbit anti-Emp1 (1:50; Abcam) overnight at 4°C. After incubation with secondary antibodies conjugated with AlexaFluor 488 and AlexaFluor 568 or AlexaFluor 647 (1:500; Invitrogen) for 1 h at room temperature, the tissues were counterstained with DAPI (Sigma-Aldrich) solution and mounted with mounting media (Vector Laboratories).

### Statistical analysis of immunostained images

Images of cortices were collected from at least three different tissues for each group, and fluorescence intensity was measured using ImageJ software (NIH). Cells with positive immunosignals over GFAP^+^ cells were counted in each image, which included ∼50 GFAP^+^ cells, sufficient for performing calculations. All of these data were presented as mean ± SE and were analyzed to determine statistically significant differences at *p* < 0.05 using the two-tailed unpaired Student’s *t* test.

## Results

### Transcription profiles of Aldh1l1-GFP+ astrocyte subpopulations demonstrate local and regional heterogeneity

To determine whether identified astrocyte subpopulations demonstrate additional heterogeneity across brain regions, we leveraged existing datasets (John Lin et al., 2017) and adopted a pairwise comparison strategy using normalized counts. Previously, five different Aldh1l1-GFP ^+^ astrocyte subpopulations were isolated from three brain regions (olfactory bulb, cortex, and brainstem). In that study, transcripts from astrocyte subpopulations and from the Aldh1l1-GFP^−^ reference population were sequenced and subpopulation-specific gene signatures were obtained (John Lin et al., 2017). To include lncRNAs in the gene signatures, we re-mapped the purified astrocyte subpopulations from different brain regions and their corresponding non-astrocyte samples to the mm10 mouse reference genome using a published pipeline (Duran et al., 2017). For gene quantification we used a comprehensive protein-coding and lncRNA annotation file including 21,948 protein-coding genes (55,252 transcripts) and 29,232 lncRNA genes (49,189 transcripts).

We implemented PCA using a gene expression matrix consisting of DE genes between regions as rows and samples with replicates as columns, yielding a 4092 × 36 matrix. We hypothesized that each PC is associated with an underlying factor regulating gene expression and that such factors might explain the variability between subpopulations and regions. PCA analysis resulted in 36 uncorrelated and orthogonal PCs that account for the variability in the gene expression matrix. The first four components explain 82.3% of the variability of these samples. To gain biological insights for the selected PCs, we plotted the samples in their transformed component space. Figure 1*A* depicts the plotted samples in the 2D-space formed by PC1 and PC2. PC1 explains 68.98% of the variability and clusters the samples into two main groups: Non-astrocyte (shapes without filling) and astrocyte (color-filled shapes) subpopulations. The majority of samples clustered well; only a few outliers appeared in the non-astrocyte sample space. Similarly, the combined effect of PC1 and PC2 is helpful for discriminating among subpopulations, as shown by the elliptical subspaces (Fig. 1*A*) that enclose shapes mostly of the same color. To cluster samples by region, we plotted PC3 and PC4, as shown in Figure 1*B*. The elliptical subspaces in the figure demonstrate the separation of samples into the olfactory bulb, cortex, and brainstem regions.

To validate the grouping of samples accomplished with PCA, we performed GSEA using DE genes ranked by the scores assigned in each PC. For PC1, the top enriched gene sets were GO_MYELIN_SHEATH and LEIN_ASTROCYTE_MARKERS (Fig. 1*C*). The top-enriched gene sets for PC2 are GO_SYNAPTIC_SIGNALING and VERHAAK_GLIOBLASTOMA_MESENCHYMAL (Fig. 1*D*). The top-enriched gene sets for PC3 are GO_MICROTUBULE_ASSOCIATED_COMPLEX and OXIDATIVE_PHOSPHORYLATION (Fig. 1*E*). Astrocyte subpopulation B (combined from all three brain regions) is mostly correlated with the mesenchymal glioblastoma gene signature, as the enriched genes in this gene set (e.g., *Scpcp1*, *Rac2*, *Blcrb*, *Mrc2*, *Cd14*, and *C5ar*, among others) are upregulated in this subpopulation.

### Differentially expressed protein-coding genes and lncRNAs depict regional subpopulation-specific gene signatures and gene sets

To obtain regional subpopulation enriched genes, we implemented a pairwise comparison strategy using normalized counts. For each brain region, we compared the diverse astrocyte subpopulations against their corresponding non-astrocyte samples. Genes with FPKM > 1, fold-change > 2, and FDR < 10% were considered to be DE. The number of unique DE genes per subpopulation and brain region are listed in Table 2. Significant numbers of DE lncRNAs contribute to the regional subpopulation gene signatures. Figure 2*A* shows a heatmap of the log2-transformed FPKM values of cortex subpopulation B-enriched genes (protein-coding and lncRNAs) compared with other subpopulations. The heatmap in Figure 2*B* illustrates the log2-transformed FPKM values of the 317 regional subpopulation upregulated lncRNA genes. After using a statistical test (unpaired two-tailed *t* test with *p* < 0.05), we observed that the fold-changes of DE lncRNAs from cortex subpopulations B–E, and olfactory bulb subpopulations A, B, C, and E had higher fold-change than their corresponding DE protein-coding genes. Extended data Figure 2-2 shows the comparison between fold-changes and *q* values of DE protein-coding and lncRNA genes from different astrocyte subpopulations and regions.

**Table 2.** Regional subpopulation-specific DE protein-coding and lncRNA genes

****	****		**Brain Region**		
**Subpopulation**	**Type**		**Olfactory bulb**	**Cortex**	**Brainstem**
PopA	Protein-coding	UP	86	5	29
		DOWN	51	0	6
	lncRNA	UP	12	1	6
		DOWN	5	0	1
PopB	Protein-coding	UP	294	690	55
		DOWN	207	324	4
	lncRNA	UP	42	138	19
		DOWN	18	23	0
PopC	Protein-coding	UP	268	271	197
		DOWN	75	94	21
	lncRNA	UP	20	15	6
		DOWN	10	11	15
PopD	Protein-coding	UP	28	5	101
		DOWN	16	1	18
	lncRNA	UP	16	4	20
		DOWN	1	1	6
PopE	Protein-coding	UP	68	43	0
		DOWN	289	18	0
	lncRNA	UP	35	13	0
		DOWN	27	2	0

**Figure 3.**
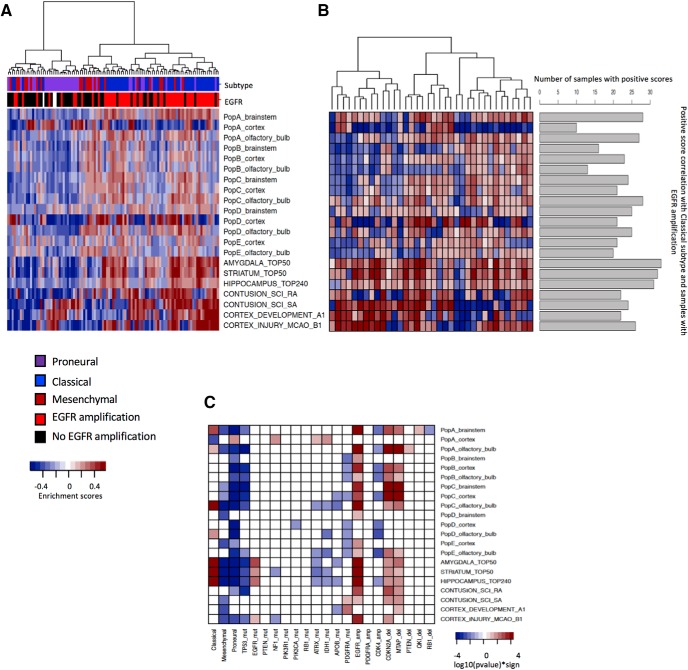
Correlation between upregulated astrocyte gene signatures and TCGA GBM samples. ***A***, Heatmap displaying the hierarchical clustering of enrichment scores obtained through GSVA. High scores indicate strong positive correlation between astrocyte gene signatures and gene expression profiles of 103 TCGA GBM samples with a tumor purity >70%. Top bars indicate tumor subtype and the presence or absence of an *EGFR* gene amplification. ***B***, Heatmap representing the enrichment scores between astrocyte gene signatures and Classical GBM samples that carry an *EGFR* gene amplification. Bar plots indicate the number of samples with positive enrichment score for each astrocyte gene signature. ***C***, Exploratory analysis of the correlation between astrocyte gene signatures and GBM samples with different subtypes, somatic mutations, and copy-number variations. The heatmap depicts the -log10-transformed *p* value of either an ANOVA comparison between GBM subtypes or a Wilcoxon rank sum test between samples carrying the selected mutations, amplifications, and deletions. For viewing clarity, only 21 representative astrocyte gene signatures are shown in each panel. Figures depicting all astrocyte gene signatures are available in Extended data Figures 3-3 and 3-4. See also Extended data Figures 3-1, 3-2, 3-3, 3-4, 3-5, 3-6.

10.1523/ENEURO.0288-18.2019.f2-3 Figure 2-3 Gene set enrichment using astrocyte subpopulation regional gene signatures. Bar plots indicate the number of genes found in the enriched gene set. The yellow line illustrates the gene set enrichment using -log10-transformed FDR. Download Figure 2-3, TIF file.

**Figure 4.**
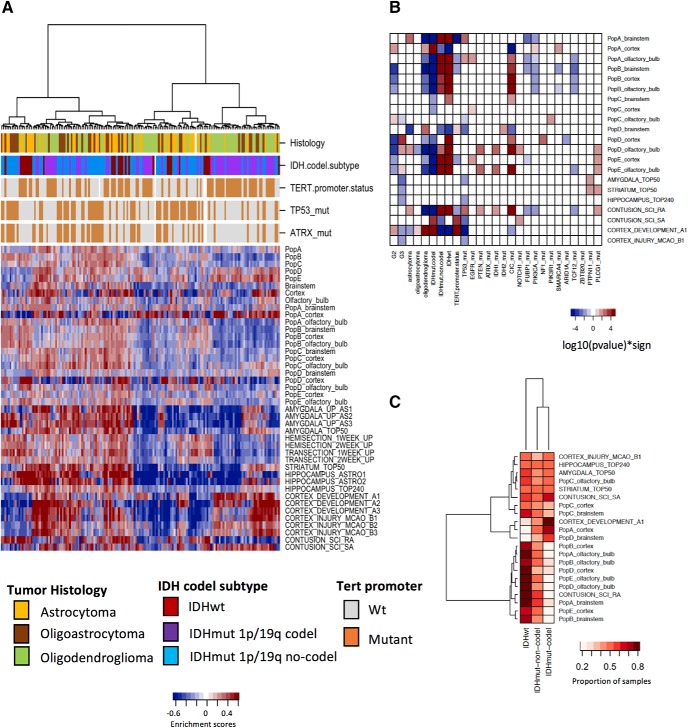
Correlation between upregulated astrocyte gene signatures and TCGA LGG samples. ***A***, Heatmap displaying the hierarchical clustering of enrichment scores obtained through GSVA. High scores indicate strong positive correlation between astrocyte gene signatures and 160 TCGA LGG samples with tumor purity >70%. Top bars indicate tumor histology and known co-occurring genomic alterations: IDH mutation combined with 1p/19q codeletion subtype (IDH.codel.subtype), mutations in TERT promoter (TERT.promoter.status), TP53 mutations, and ATRX mutations. ***B***, Exploratory analysis of the correlation between astrocyte gene signatures (*y*-axis) and LGG grades, histologic subtypes, somatic mutations, and copy-number variations (*x*-axis). The heatmap depicts the -log10-transformed *p* value of either an ANOVA comparison between LGG subtypes or a Wilcoxon rank sum test between samples carrying the selected mutations, amplifications, and deletions. ***C***, Heatmap depicting the proportion of LGG samples with specific IDH status variants that had positive enrichment scores for every astrocyte gene signature. For viewing clarity, only 21 representative astrocyte gene signatures are shown in each ***B*** and ***C***. Displays depicting complete astrocyte gene signatures are available in Extended data Figure 4-1. See also Extended data Figures 3-1, 3-2, 3-6, 4-1, 4-2, and 4-3.

10.1523/ENEURO.0288-18.2019.f2-4 Figure 2-4 Enrichment scores obtained from the gene set enrichment analysis using regional astrocyte subpopulation gene signatures. Scores represent FDR transformed into -log10 scale. The file also contains additional gene sets obtained from published studies related to neurodegenerative diseases. Gene sets were added to the MsigDB collection and used for gene set enrichment analysis. Download Figure 2-4, XLSX file.

A set of gene signature DE genes with highest expression in cortex subpopulation B compared with cortex non-astrocytes include, for example, MER proto-oncogene tyrosine kinase (*Mertk*), Ras homolog family member B (*Rhob*), and signal regulatory protein alpha (*Sirpa*; see Discussion). Similarly, a set of DE lncRNAs in cortex subpopulation B with the highest expression compared with non-astrocyte samples include, for example, *Gm37524*, *Gm3764*, and *Junos*. We used DE genes to find the enrichment of gene sets. The top five significantly enriched gene sets found in cortex subpopulation B are listed in Figure 2*C* (top) and they include “BLALOCK_ALZHEIMERS_DISEASE_UP”, “GO_CELLULAR RESPONSE_TO_CYTOKINE_STIMULUS”, “GO_CELLULAR_REPONSE_TO_STRESS”, “GO_WOUND_HEALING”, and “GO_BIOLOGICAL_ADHESION”.

A set of gene signature DE protein-coding genes with highest expression in cortex subpopulation C include, for example, glial high affinity glutamate transporter (*Slc2a1*), carboxypeptidase E (*Cpe*), and transmembrane protein 47 (*Tmem47*). DE lncRNAs enriched in cortex subpopulation C include *Gm13872*, *Gm26672*, and *Gm37885*. The top-enriched gene sets with DE genes found in cortex subpopulation C are shown in Figure 2*C* (bottom). Enriched gene sets include “GO_GENERATION_OR_PRECURSOR_METABOLITES_AND_ENERGY”, “GO_MITOCHONDRIAL_ATP_SYNTHESIS_COUPLED_PROTON_TRANSPORT”, “KEGG_PARKINSONS_DISEASE”, and “KEGG_HUNTINGTONS_DISEASE”. Extended data Figure 2-3 depicts examples of the top-enriched gene sets specific to each regional astrocyte subpopulation. The complete list of gene sets and their enrichment scores can be found in Extended data Figure 2-4.

To evaluate regional differences in the various astrocyte subpopulations, we compared all astrocyte subpopulations from one particular region against astrocyte samples from the remaining regions (FPKM > 1, fold-change > 4, *p* < 0.05). To eliminate the effect of genes also expressed in non-astrocyte subpopulations, we compared the region-specific astrocyte subpopulations against their corresponding non-astrocyte samples (FPKM > 1, fold-change > 2, *p* < 0.05). The differential expression of a gene was considered significant if it met the criteria for both comparisons (region-specific and astrocyte-specific). Table 3 shows the number of DE protein-coding and lncRNAs identified from each brain region. Figure 2*D* depicts the log2-transformed FPKM values of DE genes from all brain regions. Insulin-like growth factor 1 (*Igf1*), flavin-containing monooxygenase 1 (*Fmo1*), and Brain-specific angiogenesis inhibitor 1 (*Bai1* or *Adgrb1*). are among the top DE protein-coding genes with highest expression in olfactory bulb astrocyte subpopulations. Membrane frizzled-related protein (*Mfrp*), integrin subunit alpha 9 (*Itga9*), and transmembrane protein 218 (*Tmem218*) are within the top DE protein-coding genes in brainstem astrocyte subpopulations. Similarly, scavenger receptor class A member 3 (*Scara3*), leucine rich repeat containing 10B (*Lrrc10b*), and Cristallin mu (*Crym*) are included in the top DE protein-coding genes obtained from astrocyte subpopulations of the cortex.

**Table 3.** Number of region-specific DE protein-coding and lncRNA genes

	**Unique DE genes**				**Total DE genes**
**Brain region**	**Protein-coding**		**lncRNA**		
	UP	DOWN	UP	DOWN
Olfactory bulb	35	13	15	1	64
Cortex	6	7	3	0	16
Brainstem	21	8	5	3	37

### Upregulated astrocyte gene signatures of several subpopulations and regions correlate with glioblastoma samples

To be comprehensive in studying astrocyte heterogeneity, in addition to the five astrocyte subpopulations from three brain regions described, we also collected gene expression datasets for other astrocyte subpopulations from diverse studies in which astrocytes were purified through using single-cell microfluidics (Zeisel et al., 2015; Gokce et al., 2016; Wu et al., 2017), FACS-sorting (Rusnakova et al., 2013; Noristani et al., 2016), or laser microdissection (Hara et al., 2017). The gene signatures collected were derived from diverse subpopulations of purified astrocytes from brain (amygdala, striatum, hippocampus, and cortex), different developmental stages, and from spinal cord injury (SCI) models. Only protein-coding genes were provided in these datasets. These astrocyte subpopulation gene signatures were combined with the regional astrocyte subpopulation-specific signatures described previously, yielding a total of 42 gene signatures (Extended data Fig. 3-1) of which 17 included both protein-coding and lncRNA genes. In each gene signature profile, genes are significantly upregulated relative to the non-astrocyte sample, the remaining astrocyte subpopulations, or both. To determine the similarity among gene signatures, the Jaccard indexes were calculated and a heatmap was built (Extended data Fig. 3-2). The Jaccard index indicates the percentage of similarity because of the overlap of genes in gene signatures. The highest similarity index between the gene signatures obtained from literature and our 15 astrocyte gene signatures was 7% (olfactory bulb subpopulation C and HIPPOCAMPUS_TOP240), indicating that they are quite different. The inclusion of other datasets from the literature provided additional information. As observed in the aforementioned heatmap, similarity indexes between the majority of the gene signatures are low. Interestingly, the similarity index between cortex injury and cortex development gene signatures was the highest (Jaccard index = 0.87).

10.1523/ENEURO.0288-18.2019.f3-1 Figure 3-1 Collection of 42 astrocyte gene signatures used in downstream analysis. Download Figure 3-1, XLSX file.

10.1523/ENEURO.0288-18.2019.f3-2 Figure 3-2 Heatmap displaying the Jaccard index of genes shared between astrocyte gene signatures. Download Figure 3-2, TIF file.

We adopted the GSVA approach (Hänzelmann et al., 2013) to determine the degree of similarity between astrocyte subpopulations and glioma samples. GSVA is an unsupervised nonparametric method for evaluating the degree to which genes in a gene signature are coordinately up- or downregulated within a certain biological sample. We used GSVA with the 42 astrocyte subpopulation gene signatures as gene sets and obtained enrichment score matrices for glioblastoma and lower-grade glioma from TC GA. The enrichment score of a gene signature may be positive or negative and it provides evidence of the coordinated up or downregulation of the members of that gene signature in a particular sample. Enrichment scores are obtained without prior knowledge of the sample phenotype. In our analysis, a positive enrichment score indicates a correlation between a glioma sample and the specific astrocyte subpopulation from which the gene signature was derived.

The heatmap in Figure 3*A* shows the resulting enrichment scores of the GSVA between 103 TCGA GBM samples with a tumor purity >70% and 21 representative astrocyte gene signatures because of limited space. The enrichment score heatmap of TCGA GBM samples with all 42 astrocyte gene signatures is included in Extended data Figure 3-3. The 103 TCGA GBM samples represented 47, 22, and 34 cases of classical, mesenchymal, and proneural transcriptional subtypes, respectively, as previously defined (Wang et al., 2018). Nearly 77% (36 of 47) of the glioblastoma samples within the classical subtype exhibited EGFR gene amplification. The top five positively enriched gene signatures with the highest number of classical subtype samples carrying an EGFR amplification were amygdala_top50, striatum_top50, hippocampus_top240, PopC_olfactory_bulb, and PopA_brainstem. For each gene signature, the number of samples with positive scores are shown in Figure 3*B*. For viewing clarity, Figure 3, *B* and *C*, displays results using 21 representative astrocyte gene signatures. Please refer to Extended data Figure 3-4 for displays using all astrocyte gene signatures.

10.1523/ENEURO.0288-18.2019.f3-3 Figure 3-3 Heatmap displaying the hierarchical clustering of enrichment scores obtained through GSVA and GBM samples. High scores indicate strong positive correlation between astrocyte gene signatures and gene expression profiles of 103 TCGA GBM samples with a tumor purity >70%. Top bars indicate tumor subtype and the presence or absence of an *EGFR* gene amplification. Download Figure 3-3, TIF file.

10.1523/ENEURO.0288-18.2019.f3-4 Figure 3-4 Correlation between upregulated astrocyte gene signatures and TCGA GBM samples. ***A***, Analysis of the correlation between astrocyte gene signatures and GBM samples with different subtypes, somatic mutations, and copy-number variations. The heatmap depicts the -log10-transformed *p* value of either an ANOVA comparison between GBM subtypes or a Wilcoxon rank sum test between samples carrying the selected mutations, amplifications, and deletions. ***B***, Heatmap representing the enrichment scores between astrocyte gene signatures and Classical GBM samples that carry an *EGFR* gene amplification. Bar plots indicate the number of samples with positive enrichment score for each astrocyte gene signature. Download Figure 3-4, TIF file.

**Figure 5.**
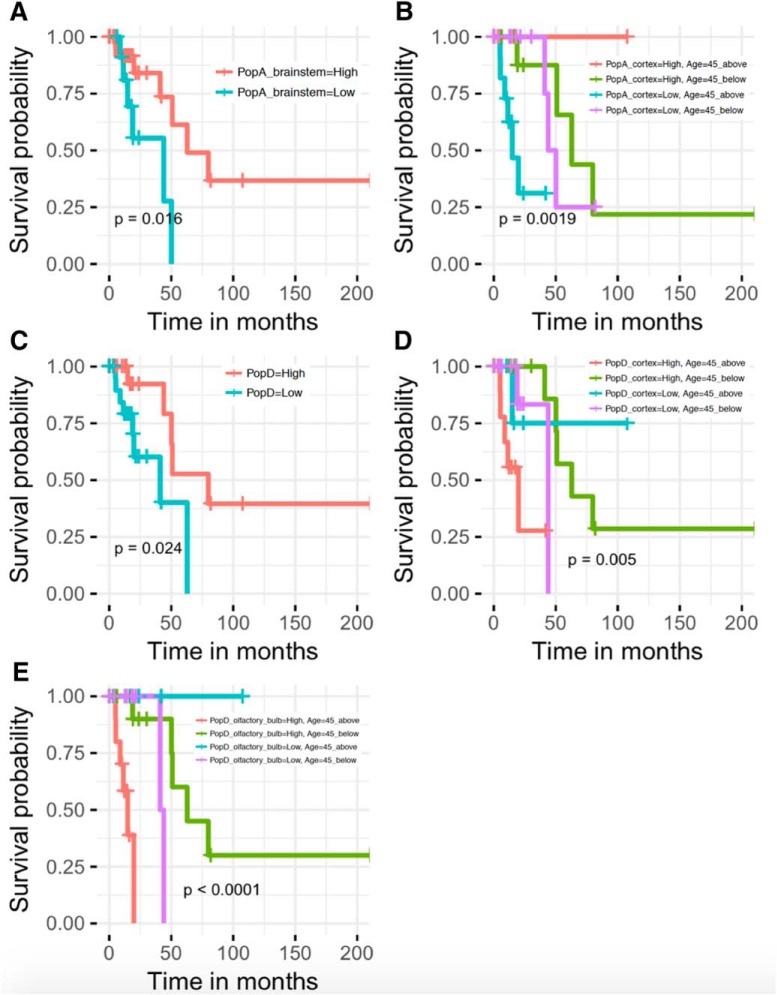
Astrocyte subpopulation gene signatures are associated with survival time differences in TCGA LGG astrocytoma patients. Figure shows Kaplan–Meier plots and log-rank test *p* values of sample groups stratified according to High and Low enrichment scores for (***A***) brainstem subpopulation A, (***B***) cortex subpopulation A and age, (***C***) subpopulation D, (***D***) cortex subpopulation D and age, and (***E***) olfactory bulb subpopulation D and age.

10.1523/ENEURO.0288-18.2019.f3-5 Figure 3-5 Correlation of region-specific astrocyte subpopulation enrichment scores with 103 TCGA Glioblastoma samples with tumor purity >70%. A Wilcoxon rank sum test was used to compare the distribution of enrichment scores between samples with and without amplification of EGFR. AMP, Amplification of EGFR; No AMP, no amplification of EGFR found. ***p < 0.001, **p < 0.01, *p < 0.05. Download Figure 3-5, TIF file.

**Figure 6.**
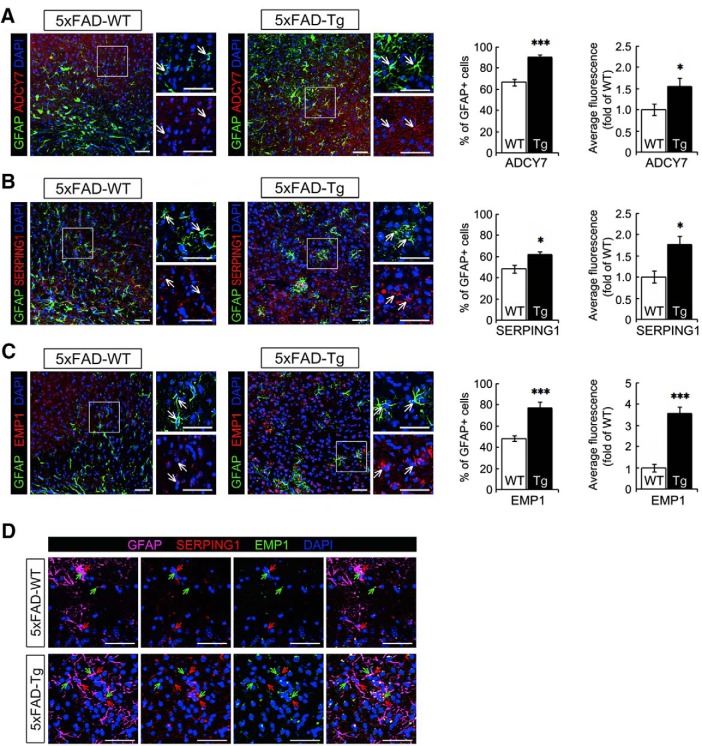
Protein expression of candidate genes in the brain tissues from the 5xFAD Alzheimer’s disease mouse model. Brain tissues collected from 5xFAD mouse models were prepared for immunofluorescence using anti-GFAP (green) and either (***A***) anti-Adcy7 (***B***) anti-Serping1, or (***C***) anti-Emp1. The regions outlined with a square are displayed at higher-magnification on the right, and the arrows point to the same cells for comparison in the fluorescent images. For the astrocytes in the inner layer of cortex (the area near the corpus callosum), the proportion of GFAP^+^ cells displaying red fluorescence was calculated, and the intensity of red fluorescence overlapping with GFAP signals was measured. Two-tailed unpaired Student’s *t* tests were used to compare AD samples to the wild-type group. ***D***, Coimmunostaining of cortical brain tissues was performed with anti-GFAP, anti-Serping1, and anti-Emp1. *p < 0.05, **p < 0.01, ***p < 0.001. Scale bars (in ***A***–***D***), 50 μm. See Extended data Figures 6-1, 6-2, and 6-3.

10.1523/ENEURO.0288-18.2019.f3-6 Figure 3-6 GSVA enrichment scores obtained between the 42 astrocyte subpopulation gene signatures and TCGA GBM or LGG samples. Correlation matrices of -log10 transformed *p* values are also included. Download Figure 3-6, XLSX file.

To evaluate whether the differences among groups of samples with different genomic features (such as subtype, mutations, and copy-number variations) could be explained with GSVA enrichment scores, ANOVA and Wilcoxon rank sum tests were performed. Each genomic feature was correlated to all gene signatures by comparing enrichment scores between samples grouped according to the different levels of the genomic features. The correlation matrix of genomic features and 21 representative signature scores is depicted in Figure 3*C*. Correlations were deemed significant with a *p* < 0.05. Significant positive correlations with the Classical GBM subtype samples were found in 11 out of 42 astrocyte gene signatures including brainstem subpopulation A, olfactory bulb (subpopulations A, C, and D), amygdala, striatum, and hippocampus. Classical GBM subtype had the highest number of positively correlated astrocyte gene signatures, consistent with a previous report (Verhaak et al., 2010). Mesenchymal and proneural GBM subtypes had either negative or no significant correlations. No significant positive correlations were found for GBM samples with TP53 mutations.

A large proportion of astrocyte gene signatures were significantly positively correlated with samples carrying amplifications of the gene encoding EGFR (62%, 26 of 42) and deletions of the genes encoding CDKN2A and MTAP (50 and 45%). Gene signatures of subpopulations A, B, and C, brainstem (subpopulations A, C), olfactory bulb (subpopulations A, B, C, E), cortex (subpopulations B, C), amygdala, striatum, hippocampus, reactive astrocytes (RAs), and scar-forming astrocytes (SAs) in SCI had significant positive correlations with GBM samples with a combined set of genomic features that included amplification of the EGFR gene and deletions of the CDKN2A and MTAP genes. Boxplots depicting the correlation of regional astrocyte subpopulation gene signatures with GBM samples with EGFR amplification is included in Extended data Figure 3-5. Gene signatures of subpopulations A and C from brainstem and olfactory bulb, and from cortex subpopulation B, were positively correlated with EGFR amplifications with *p* < 0.001.

### Upregulated astrocyte gene signatures in several subpopulations and regions correlate with LGG samples

A total of 160 LGG samples (tumor purity >70%) from TCGA were used for analysis of correlation with astrocyte gene signatures. The histologic classifications of the LGG samples used included 44 astrocytomas, 45 oligoastrocytomas, and 70 oligodendrogliomas. Figure 4*A* shows the enrichment scores obtained using GSVA. The upper section of the heatmap shows the classification of different co-ocurring genomic alterations previously shown to be related to glioma histology (Cancer Genome Atlas Research Network et al., 2015). Two groups are formed corresponding mostly of oligodendroglioma (right side) and a mixture enriched in astrocytoma and oligoastrocytoma histologies (left side). As previously demonstrated (Cancer Genome Atlas Research Network et al., 2015), the mixture of astrocytoma and oligoastrocytoma samples is characterized modestly by mutations of IDH without codeletions of 1p/19q loci, TP53 mutations, and ATRX mutations. The oligodendroglioma group is enriched in samples carrying mutations of *IDH* gene combined with codeletions of 1p/19q loci, and mutations in the TERT promoter. A cluster of positive high enrichment scores between LGG samples and astrocyte gene signatures is observed on the left side of the heatmap corresponding mostly to the mixture of astrocytoma and oligoastrocytoma histologies. Brainstem subpopulation A, olfactory bulb subpopulation D, and RA SCI astrocyte gene signatures were significantly correlated with astrocytoma LGG samples. Amygdala (AS1–AS3) and cortex development gene signatures were correlated with oligoastrocytoma histology. Subpopulations D and E, cortex subpopulation A, brainstem subpopulation D, cortex injury (B2), and cortex development gene signatures were correlated with oligodendroglioma. The heatmap in Figure 4*B* shows the correlation matrix between 21 astrocyte gene signatures and LGG samples with diverse genomic features because of limited space. Extended data Figure 4-1 includes a heatmap representing the correlation between LGG samples and all 42 astrocyte gene signatures. Approximately fourteen percent and 7% of the 42 astrocyte gene signatures were positively correlated with grade 2 and grade 3 LGG samples, respectively.

10.1523/ENEURO.0288-18.2019.f4-1 Figure 4-1 Correlation between upregulated astrocyte gene signatures and TCGA LGG samples. ***A***, Analysis of the correlation between astrocyte gene signatures (*y*-axis) and LGG grades, histological subtypes, somatic mutations, and copy-number variations (*x*-axis). The heatmap depicts the -log10-transformed *p* value of either an ANOVA comparison between LGG subtypes or a Wilcoxon rank sum test between samples carrying the selected mutations, amplifications, and deletions. ***B***, Heatmap depicting the proportion of LGG samples with specific IDH status variants that had positive enrichment scores for every astrocyte gene signature. Download Figure 4-1, TIF file.

10.1523/ENEURO.0288-18.2019.f4-2 Figure 4-2 Heatmaps depicting GSVA enrichment score matrices between astrocyte gene signatures and TCGA LGG samples. Samples with (***A***) wild-type IDH1 (***B***) mutant IDH1 with 1p/19q codeletion, and (***C***) mutant IDH1 without 1p/19q codeletion are compared. Download Figure 4-2, TIF file.

10.1523/ENEURO.0288-18.2019.f4-3 Figure 4-3 Heatmaps depicting GSVA enrichment score matrices between astrocyte gene signatures and TCGA LGG samples with different features. Samples with (***A***) astrocytoma histology and wild-type IDH1or mutant IDH1 without 1p/19q codeletion, and (***B***) oligodendroglioma histology and mutant IDH1 with 1p/19q codeletion, mutant TERT promoter, and mutations in CIC gene are compared. Download Figure 4-3, TIF file.

Mutation in the gene encoding IDH1 or IDH2 and complete deletion of both the short arm of chromosome 1 and the long arm of chromosome 19 (1p/19q codeletion) are markers frequently used in clinical practice for classification of LGG samples. Both are indicators of favorable prognosis. We used IDH status as a genomic feature with the following categories: wild-type IDH, mutant IDH with codeletion of 1p/19q, and mutant IDH with no codeletion of 1p/19q. ∼45% and 40% of the gene signatures were positively correlated with wild-type IDH samples and mutant IDH samples with no 1p/19q codeletion, respectively. Subpopulation B, brainstem (subpopulations A, B), olfactory bulb (subpopulations A, B, D, E), cortex (subpopulations B, E), hemisection, transection, hippocampus (ASTRO1), and RA SCI astrocyte gene signatures correlated significantly with both wild-type IDH and mutant IDH samples with no1p/19q codeletion. Figure 4*C* shows a heatmap displaying the proportion of samples featuring each IDH status variant with positive enrichment scores for 21 representative astrocyte gene signatures (Extended data Fig. 4-1 displays all 42 astrocyte gene signatures). Extended data Figure 4-2*A*–*C* shows the enrichment score matrices obtained with each IDH status variant. For wild-type IDH, olfactory bulb (subpopulations A, B, D, and E), cortex (subpopulations B and D), and brainstem subpopulation A gene signatures have positive enrichment scores with the highest proportion of samples. Mutant IDH samples without the 1p/19q codeletion had high enrichment scores with amygdala gene signatures (AS1–AS3). Mutant IDH samples carrying the 1p/19q codeletion had high enrichment scores with subpopulations D and E, cortex subpopulation A, cortex injury (B2), cortex development (A1, A2), and SA SCI gene signatures. Table 4 shows the distribution of histology class and IDH status of the 160 LGG samples used in our analysis. The wild-type IDH and mutant IDH without 1p/19q codeletion groups consisted mostly of astrocytomas (44 and 45%), whereas mutant IDH samples with the 1p/19q codeletion were mostly oligodendrogliomas (80%). Extended data Figure 4-3*A* shows the enrichment scores for LGG samples with an astrocytic histology and wild-type IDH or mutant IDH with no 1p/19q codeletion. Brainstem subpopulation A and olfactory bulb subpopulations D and E are among the top five gene sets with positive enrichment scores that have the highest number of samples with the aforementioned features.

**Table 4.** Histologic classification of LGG samples with tumor purity >70% used in this study in each IDH status group

**Histological class**	**IDH wild-type (27 samples)**	**Mutant IDH + no 1p/19q codeletion (71 samples)**	**Mutant IDH + 1p/19q codeletion (61 samples)**
Astrocytoma	12	32	0
Oligoastrocytoma	8	25	12
Oligodendroglioma	7	13	49

Mutations in the promoter of the gene encoding TERT were positively correlated with astrocyte gene signatures (cortex development, cortex injury, SA SCI) in 12% of the LGG samples. Positive correlations were found in 36% of the gene signatures for LGG samples carrying mutations in the CIC gene. Astrocyte subpopulation gene signatures (PopD, PopE, Cortex, and PopA cortex) clustered a subset of LGG samples into oligodendrogliomas with IDH mutation and 1p/19q codeletion, TERT promoter mutations, and *CIC* gene mutations. The heatmap in Extended data Figure 4-3*B* contains the enrichment scores of samples with an oligodendroglioma histology, mutant TERT promoter, mutant *CIC* gene, and mutant *IDH* gene combined with the 1p/19q codeletion.

Kaplan–Meier survival plots are commonly used to assess treatment efficacy in clinical trials. To determine whether astrocyte gene signatures are correlated with the survival of glioma patients, we performed survival analysis using the survival R library (Therneau, 2015). Kaplan–Meier estimates of overall survival among patients who had LGG samples with an astrocytoma histology were obtained for each gene signature. We grouped samples into negative (Low) and positive (High) enrichment scores, with and without age as a factor, as described in Materials and Methods. Figure 5*A*–*E* depicts significant (*p* < 0.05) survival plots with distinct astrocyte gene signatures. Better prognosis (without an age effect) was observed for patients with astrocytoma LGG samples correlated with brainstem subpopulation A (Fig. 5*A*) and subpopulation D (Fig. 5*C*) gene signatures. Age had a greater effect than enrichment score on the probability of survival for patients with astrocytoma LGG samples correlated with cortex subpopulation A (Fig. 5*B*), cortex subpopulation D (Fig. 5*D*), or olfactory bulb subpopulation D (Fig. 5*E*). Astrocytoma LGG patients older than 45 years with profiles positively associated with subpopulation D in cortex (Fig. 5*D*) and olfactory bulb (Fig. 5*E*) had worse prognoses, as did LGG patients older than 45 years with Low enrichment scores for profiles associated with cortex subpopulation A (Fig. 5*B*).

To test whether the observed differences in survival (Fig. 5) can be explained by specific genetic mutations associated with subtypes of LGG tumors, we performed statistical analysis. We used IDH mutation, a common previously known mutation of glioma samples. We obtained 2 × 2 contingency tables with the number of LGG samples classified as High and Low (correlation with gene signatures) and as “IDHwt” or “Mutated_IDH”. Then we used Fisher’s test to determine whether the proportion of samples classified as High or Low was different depending on the number of samples with and without IDH mutations. We found that for some gene signatures (brainstem subpopulation A, cortex subpopulation A, and cortex subpopulation D) the *p* value of Fisher’s test was not significant (*p* values = 0.4606, 0.09365, and 0.08331, respectively), therefore there is no statistical evidence to demonstrate that there are differences in the enrichment scores between IDHwt and mutated IDH. However, for subpopulation D and olfactory bulb subpopulation D, the *p* values were 0.04211 and 0.001592, respectively, thus, the correlation between survival and enrichment scores among these samples could possibly be affected by mutations of IDH.

### Astrocyte subpopulation gene signatures correlate with neurodegenerative disease gene sets

In addition to correlating the signatures of astrocyte subpopulations with glioma samples, we also investigated their correlation with neurodegenerative diseases. We collected gene sets derived from neurodegenerative disease studies (Extended data Fig. 2-4) from the literature and combined them with related gene sets from the MsigDB database (Liberzon et al., 2011). Using a hypergeometric test, the DE genes from each regional astrocyte subpopulation were analyzed to identify the enrichment of gene sets related to neurodegenerative diseases including AD, Huntington's disease (HD), and Parkinson's disease (PD). Several neurodegenerative disease gene sets were found to be enriched using regional subpopulation gene signatures at FDR < 0.05. As an example, “BLALOCK ALZHEIMERS DISEASE UP” was significantly enriched with 97 upregulated genes from the cortex subpopulation B gene signature. Extended data Figure 6-1*A* shows a violin plot representing the normalized counts of the 97 upregulated genes from the cortex subpopulation B gene signature. The gene expression of these 97 genes is also high in the subpopulation B profiles of brainstem and olfactory bulb, because of their inherent similarity. The “LABADORF_HUNTINGTONS_UP” gene set was significantly enriched with 89 DE genes from the cortex subpopulation B gene signature (Extended data Fig. 6-1*B*). DE genes from cortex subpopulation C were enriched for “KEGG HUNTINGTONS DISEASE” and “KEGG PARKINSONS DISEASE” gene sets, with 13 and 11 genes respectively (Extended data Fig. 6-1*C*). The complete list of significantly enriched neurodegenerative disease gene sets can be found in Extended data Figure 6-3.

10.1523/ENEURO.0288-18.2019.f6-1 Figure 6-1 Violin plots representing normalized count distributions. Normalized count distributions of (***A***) the 97 upregulated genes from the cortex subpopulation B gene signature found in the Alzheimer’s disease gene set (***B***) the 87 upregulated genes from the cortex subpopulation B gene signature found in the Huntington’s disease gene set, and (***C***) the 11 upregulated genes from the cortex subpopulation C gene signature found in the Parkinson’s disease gene set are shown in violin plots. Download Figure 6-1, TIF file.

### The expression of astrocyte genes enriched in the Alzheimer’s disease gene set was validated using immunostaining in mouse disease models

Significant enrichment of the AD gene set was found in DE genes from cortex astrocyte subpopulation B. For validation, we selected three genes with gene expression fold-change >4 compared with the corresponding non-astrocyte samples. We used two mouse models of AD: 5xFAD (Polito et al., 2014) and NLGF (Saito et al., 2014). AD pathology was confirmed in the cortex as an increase in GFAP^+^ astrocytes as well as detectable hypertrophy, as shown in Extended data Figure 6-2*A*. Because subpopulation B astrocytes reside mainly in the inner cortex (John Lin et al., 2017), tissue sections from these brain regions were analyzed. The protein expression of *Adcy7*, *Serping1*, and *Emp1* genes was analyzed using immunofluorescence in inner cortex brain tissue sections. Expression of Adcy7 protein was identified in 60% of WT cortical astrocytes. In contrast, >80% of cortical astrocytes from 5xFAD mice models expressed Adcy7 protein with an average fold-change of 1.5 ±0.18 and *p* < 0.05 (Fig. 6*A*). Protein expression of the *Serping1* gene was found in ∼50% cortical WT astrocytes and was upregulated in AD with a fold-change of 1.72 ± 0.19 and *p*- < 0.05 (Fig. 6*B*). Similarly, nearly 50 and 80% of cortical WT and AD astrocytes, respectively, expressed the Emp1 protein, which was upregulated 3.57 ± 0.27 times with *p* < 0.001 (Fig. 6*C*).

10.1523/ENEURO.0288-18.2019.f6-2 Figure 6-2 Protein expression of candidate genes in the brain tissues from the NLGF Alzheimer’s disease mouse model. ***A***, AD pathology was confirmed in the cortex as an increase in GFAP^+^ astrocytes as well as detectable hypertrophy in 5xFAD and NLGF AD mouse models. Brain tissues collected from NLGF mouse models were prepared for immunofluorescence using anti-GFAP (green) and either (***B***) anti-Adcy7 (***C***) anti-Serping1, or (***D***) anti-Emp1. The regions outlined with a square are displayed at higher-magnification on the right, and the arrows point to the same cells for comparison in the fluorescent images. For the astrocytes in the inner layer of cortex (the area near the corpus callosum), the proportion of GFAP^+^ cells displaying red fluorescence was calculated, and the intensity of red fluorescence overlapping with GFAP signals was measured. Two-tailed unpaired Student’s *t* tests were used to compare AD samples to the wild-type group. ***E***, Coimmunostaining of cortical brain tissues was performed with anti-GFAP, anti-Serping1, and anti-Emp1. *p < 0.05, **p < 0.01, ***p < 0.001. Scale bar (in ***A***–***D***), 50 μm. Download Figure 6-2, TIF file.

10.1523/ENEURO.0288-18.2019.f6-3 Figure 6-3 Significantly enriched gene sets related to neurodegenerative diseases (AD, HD, and PD). Gene set enrichment was obtained with differentially expressed genes specific for each regional astrocyte subpopulation. Download Figure 6-3, XLSX file.

Microarray studies have reported two types of reactive astrocytes, A1 and A2 astrocytes, that are induced after neuroinflammation and ischemia, respectively (Zamanian et al., 2012; Liddelow et al., 2017). Each type of astrocyte has its own reactive profile with differences in transcriptome, morphology, and function. Since Serping1 protein is one of the known markers for A1 reactive astrocytes and Emp1 protein is one of the known markers for A2, we evaluated their combined expression in cortical astrocytes. Figure 6*D* shows the single and combined fluorescence images in both WT and 5xFAD cortical tissues. The immunostaining results from the NLGF AD mouse model are included in Extended data Figure 6-2*B*–*E* and were similar to results from the 5xFAD model.

## Discussion

Currently there is no effective treatment for glioma or for neurodegenerative diseases such as AD, PD, and HD, because our understanding of their pathophysiology is far from complete. Mounting evidence indicates that astrocytes are involved in various neurologic diseases. Moreover, it is now known that astrocytes are functionally diverse and respond differently to pathologic conditions.

Astrocytes are heterogeneous in morphology, developmental origin, gene expression profile, and physiologic properties (Zhang and Barres, 2010). Previous research demonstrated the existence of at least five different astrocyte subpopulations from three brain regions (olfactory bulb, cortex, and brainstem; John Lin et al., 2017). Analogous astrocyte subpopulations were found in human and mouse glioma. Astrocyte subpopulation C and its analog in glioma were enriched in the expression of genes associated with synapse formation and epilepsy. In the present study, we expanded the genomic analyses of these heterogeneous astrocyte subpopulations to include the expression of lncRNA genes and the identification of gene signatures enriched in astrocyte subpopulations within each brain region. We found that although astrocyte subpopulations share similarities across regions, there are interesting differences among them in gene expression. Astrocyte subpopulations display distinct lncRNA gene expression patterns among brain regions highlighting the contribution of lncRNAs to the different astrocyte gene signatures and to potential regulatory functions.

We selected subsets of DE protein-coding genes from astrocyte gene signatures to gain insight into the functions of astrocyte subpopulations in different brain regions. Highly expressed DE protein-coding genes enriched in cortex subpopulation B included *Mertk* and *Sirpa*, both known as synaptic phagocytic genes. MERTK interacts with the integrin pathway regulating CdKII/DOCK180/Rac1 modules, which control the rearrangement of the actin cytoskeleton during phagocytosis (Wu et al., 2005). Previous research has demonstrated that astrocytes in both the developing and the adult CNS contribute to neural circuit remodeling by phagocytosing synapses via the MEGF10 and MERTK pathways in response to neural activity (Chung et al., 2013). However, although *Mertk* is still expressed in mature astrocytes, synaptic phagocytosis declines in adult CNS (Chung et al., 2013); thus inhibitory mechanisms might be upregulated. SIRPA membrane receptor recognizes CD47 and on binding inhibits synapse phagocytosis (Barclay and van den Berg, 2014). Researchers have found that the expression of *Sirpa* membrane receptor is also upregulated in mature astrocytes (Sloan et al., 2017). In the previous research, cortex subpopulation B was found at a later stage during cortical development compared with subpopulations A, C, and E (John Lin et al., 2017). MERTK and SIRPA potentially regulate synaptic density in cortex subpopulation B during late cortical development.

*Slc1a2* and *Cpe* are among the highly expressed DE protein coding genes found in cortex subpopulation C samples. SLC1A2 is a glial amino acid transporter that plays a major role in synaptic glutamate clearance. The dysfunction of SLC1A2 (commonly observed in neurodegenerative diseases) causes elevated levels of glutamate, which yield neuronal damage (Lin et al., 2012). In a previous study, cocultures with neurons demonstrated that cortex subpopulation C astrocytes significantly enhanced synapse formation compared with subpopulation A or bulk astrocytes (John Lin et al., 2017). Further functional studies are required to determine whether the upregulation of *Slc1a2* is related to the enhancement of synapse function. Another DE protein coding gene found with high expression in cortex subpopulation C is *Cpe*. CPE is a peptidase which may also function as a neurotrophic factor promoting neuronal survival. Interestingly, CPE has been found to be secreted by cultured astrocytes (Klein et al., 1992) and its overexpression in a mouse model of glioma was found to mitigate cell migration (Armento et al., 2017). Through transwell assays, astrocyte cortex subpopulation C was identified as having less migratory potential than subpopulation A (John Lin et al., 2017). Our results suggest a potential link between the upregulation of CPE peptidase and cell migration. Overall, astrocyte gene signatures may be used to gain insight into their region and subpopulation-specific functions.

PCA and GSEA analyses yielded significantly enriched gene sets relevant to functions in specific astrocyte subpopulations and brain regions. Through PCA, we found that PC1 separated samples into non-astrocytes and astrocyte samples, whereas PC2 clustered samples according to subpopulation. The gene set GO_MYELIN_SHEATH was among the top enriched sets using PC1 scores. The enrichment of this gene set is likely because of the upregulation of astrocyte-derived factors which support oligodendrocyte myelination in the CNS. Together, PC1 and PC2 captured nearly 69% of the variability. PC3 and PC4 further separated samples according to brain region. Our results demonstrate the heterogeneity in astrocyte gene expression profiles because of subpopulation and brain region.

Many genes become upregulated in astrocytes in response to injuries and diseases through a transformation called *reactive astrogliosis*. As a functionally diverse cell population, the responses of astrocytes to injury and disease are equally diverse. Zamanian et al. (2012) found that astrocytes exist in at least two reactive states, A1 (inflammatory) and A2 (ischemic). A1 neuroinflammatory reactive astrocytes exhibit upregulation of complement cascade genes that leads to loss of synapses. In contrast, A2 ischemic reactive astrocytes display upregulation of neurotrophic factors such as thrombospondins that promote synapse recovery and repair (Zamanian et al., 2012). A1 reactive astrocytes have been found in postmortem brain tissue from patients with neurodegenerative diseases (AD, HD, PD), amyotrophic lateral sclerosis, and multiple sclerosis (Liddelow et al., 2017). Furthermore, reactive astrocytes have also been found neighboring tumor cells and might enhance their malignancy by inducing cell proliferation and migration (Le et al., 2003; Biasoli et al., 2014). A better understanding of the heterogeneity of astrocytes and their potential involvement in and contributions to different diseases is needed to promote the development of treatments targeting specific astrocyte subpopulations.

Altered astrocyte function is increasingly recognized as a factor contributing to glioma and a number of neurodegenerative diseases. Given the glial-like histopathology of glioma and its underlying cellular diversity, certain types of glioma might be composed of malignant analogues of astrocyte subpopulations (Patel et al., 2014; Laug et al., 2018). Thus, we surveyed multiple previously published astrocyte subpopulation datasets and correlated our collection of astrocyte subpopulation gene signatures with those of various subtypes of glioma and neurodegenerative diseases. Our results indicate that distinct astrocyte gene signatures are correlated to glioma samples with specific genomic features and somatic mutations. We correlated astrocyte gene signatures with copy-number variations that are known to play a role in tumor proliferation. For example, amplifications of the EGFR gene are known to lead to tumor growth through enhancement of cell proliferation. MTAP and CDKN2A genes are frequently deleted in human cancers (Mavrakis et al., 2016). A total of 18 astrocyte gene signatures were significantly correlated with GBM samples harboring a combination of copy-number variations including EGFR amplification, CDKN2A deletion, and MTAP deletion. Subpopulation C from all regions, subpopulation A (brainstem and olfactory bulb), and subpopulation B (cortex and olfactory bulb) were among the aforementioned gene signatures.

We also correlated astrocyte gene signatures with LGG samples. The correlation between astrocyte gene signatures and LGG samples improved when IDH-1p/19q status was considered than when histologic class was accounted for, consistent with a previous report (Cancer Genome Atlas Research Network et al., 2015). Our results indicate that more than half of the gene signatures were positively correlated with samples classified either as wild-type or mutated IDH with no 1p/19q codeletion. The high positive correlation of gene profiles with wild-type and mutant IDH samples without the 1p/19q codeletion is most likely because of the astrocyte origin of these gene signatures. Interestingly, most of the astrocyte subpopulations from the olfactory bulb (A, B, D, E) were strongly correlated with wild-type IDH. It is possible that astrocytomas acquire a signature that is most similar to olfactory bulb astrocytes. Our results also show that amygdala gene signatures (AS1–AS3) were strongly correlated with samples with mutant IDH and no 1p/19q codeletion. The gene signatures of subpopulations D and E, cortex subpopulation A, cortex development (A1 and A2), and cortex injury (B2) were most highly correlated with LGG samples bearing the IDH mutation and the 1p/19q codeletion; they are also positively associated with oligodendroglioma histology. This is very interesting because it provides potential links between key genomic mutations and the resultant cellular phenotypes. Although the gene signatures were derived from astrocyte subpopulations, the correlation with oligodendroglioma samples might indicate that low-grade oligodendrogliomas contain proliferating glial progenitor cells that dedifferentiated from astrocyte subpopulations, consistent with a previous report (Dai, 2001).

Survival analysis was conducted to determine whether enrichment scores of astrocyte signatures are correlated with the survival probability of patients. Kaplan–Meier plots suggest that patients with an astrocytic LGG and a gene profile similar to that of astrocyte subpopulation A in brainstem are not correlated with mutations of IDH and have a better prognosis. On the other hand, the survival of LGG patients associated with subpopulation D and olfactory bulb subpopulation D gene signatures could possibly be affected by mutations of IDH correlated with subtypes of these LGG samples.

The gene expression correlations found between astrocyte gene signatures and TCGA GBM/LGG samples suggest that certain subtypes of glioma could potentially originate from specific astrocyte subpopulations. Future experimentation will help testing the gene expression correlations identified in this work to advance the understanding and treatment of brain tumors. For example, interesting work has been done to generate RNA-seq transcriptional profiles from patient-derived glioma stem cells (Jin et al., 2017; Park et al., 2017; Puchalski et al., 2018). Finding the enrichment of astrocyte subpopulation and region gene signatures in patient-derived glioma stem cell models may pinpoint astrocyte subpopulations which might be involved in gliomagenesis.

In addition to glioma, increasing evidence suggests that astrocytes dysfunction is also involved in neurodegenerative diseases (Sofroniew and Vinters, 2010). Through GSEA, we found that cortex and olfactory bulb gene profiles of subpopulations B and C were enriched in AD, PD, and HD. GO terms related to mitochondrial respiratory chain complex were significantly enriched with genes in the cortex region astrocyte gene signature. As a result, astrocyte subpopulations in the cortex might be more vulnerable to mitochondrial dysfunction that could be associated with neurodegenerative diseases. The AD gene set was enriched with DE genes from cortex subpopulation B. Immunostaining assays validated the protein expression of three highly upregulated genes in cortex subpopulation B in two AD mouse models. Inner cortices of AD mice have significantly more GFAP ^+^ astrocytes than do wild-type mouse brains. Because subpopulation B astrocytes reside mostly in the inner cortex, we assumed that cortical GFAP^+^ cells belong mainly to this subpopulation. GFAP ^+^ astrocytes in AD cortices also exhibit significantly increased Adcy7 protein expression. Adcy7 is a membrane-bound enzyme that catalyzes the formation of cAMP (cAMP) from ATP (Crown Human Genome Center, 1997; Safran et al., 2003). Increased immunostaining of cAMP coimmunolocalizes with beta-amyloid proteins in cerebral cortical vessels of AD patients (Martínez et al., 2001). Furthermore, significantly elevated levels of cAMP have been found in CSFs of AD patients (Martínez et al., 1999). The above evidence suggests a potential role of cAMP in enhancing the progression of AD. Our results indicate that cortex subpopulation B might be involved in AD development as a response to an insult or stimuli. One of the possible mechanisms by which cortex subpopulation B might contribute to the progression of AD is through the upregulation of Adcy7, which would subsequently enhance the synthesis of cAMP from ATP. Studies involving a decrease in the expression of Adcy7 in cortex subpopulation B astrocytes from AD mice are required to better understand the underlying pathways and mechanisms of AD development.

In summary, we have demonstrated that astrocyte subpopulations from diverse brain regions have unique gene signatures for both protein-coding and lncRNA genes. Regional astrocyte subpopulation gene signatures are enriched in different functional gene sets, indicating their heterogeneity. We also obtained from the literature a comprehensive collection of gene signatures of purified astrocyte subpopulations from diverse developmental stages, brain regions, and conditions. Through analyses of gene set enrichment, we found that gene signatures of specific astrocyte subpopulations correlated with distinct glioma subtypes with unique genomic alterations. Additionally, we demonstrated the association of different astrocyte gene signatures with neurodegenerative diseases. We validated the upregulated protein expression of a set of genes in cortical astrocytes in two mouse models of AD. Thus, our analysis indicates that certain subtypes of glioma and neurodegenerative diseases are correlated to specific astrocyte subpopulations. Targeting specific astrocyte subpopulations could present a novel strategy for treating these devastating neurologic diseases.

## Synthesis

Reviewing Editor: Chay Kuo, Duke University Decisions are customarily a result of the Reviewing Editor and the peer reviewers coming together and discussing their recommendations until a consensus is reached. When revisions are invited, a fact-based synthesis statement explaining their decision and outlining what is needed to prepare a revision will be listed below. The following reviewer(s) agreed to reveal their identity: NONE.

Reviewer #1

This is a re-submission. The authors have done a good work in revising the manuscript, primarily by providing additional information (e.g., lncRNA) and rewording result/discussion (e.g., removing speculative statements). This work, relying heavily on analysis of gene expression signature/correlation and is descriptive in nature, provides detailed information of gene expression profiles in sub-groups of astrocytes, which are useful to the field of astrocyte biology. the link of sub-group astrocyte's gene expression signatures to different subtypes of grain tumors are potentially interesting. Removal of multiple highly speculative statements is an improvement.

Two more specific comments:

(1) The discussion of ITGAV is quite speculative - why is this particular important, and what is its relationship to EGFR, based on the gene expression correlation?

(2) The discussion can be improved by including more specific strategies/designs for ways to test gene expression correlations identified in this work in term of advancing understand/treatment of brain tumors. For example, how these can help identifying brain tumor cells of origins, their biology and treatments.

Reviewer #2

The authors have addressed many of concerns raised in the first review. However, there are some issues to be clarified.

1. The authors classification of a population of astrocytes with the gene set enrichment in GO_MYELIN_SHEATH is intriguing (Fig. 1C). They suggest that it is because astrocytes promote myelination. The authors may likely mix the astrocyte function with astrocyte cell identity. The claim of the ‘MYELIN_SHEATH’-enriched astrocyte subpopulation needs to be further clarified or validated.

2. The authors indicate that the previous classification of astrocyte subpopulations was ‘supervised’. The authors should clarify how they were ‘supervised’ in the previous study. Also, are the same parameters used for previous ‘supervised’ and current ‘unsupervised’ analyses?

3. Instead of listing of gene ontology terms for astrocyte subpopulations, it would be more informative to present a table listing individual gene expression levels across different astrocyte subpopulations, so that the one would be able to search for the expression of a gene of interest in each identified astrocyte subpopulation.
