# Supplementary figures and images for: Brain Region-Specific Gene Signatures Revealed by Distinct Astrocyte Subpopulations Unveil Links to Glioma and Neurodegenerative Diseases
Source: eNeuro. 2019 Apr 2;6(2):ENEURO.0288-18.2019. doi: 10.1523/ENEURO.0288-18.2019 (PMC6449165; doi:10.1523/ENEURO.0288-18.2019)

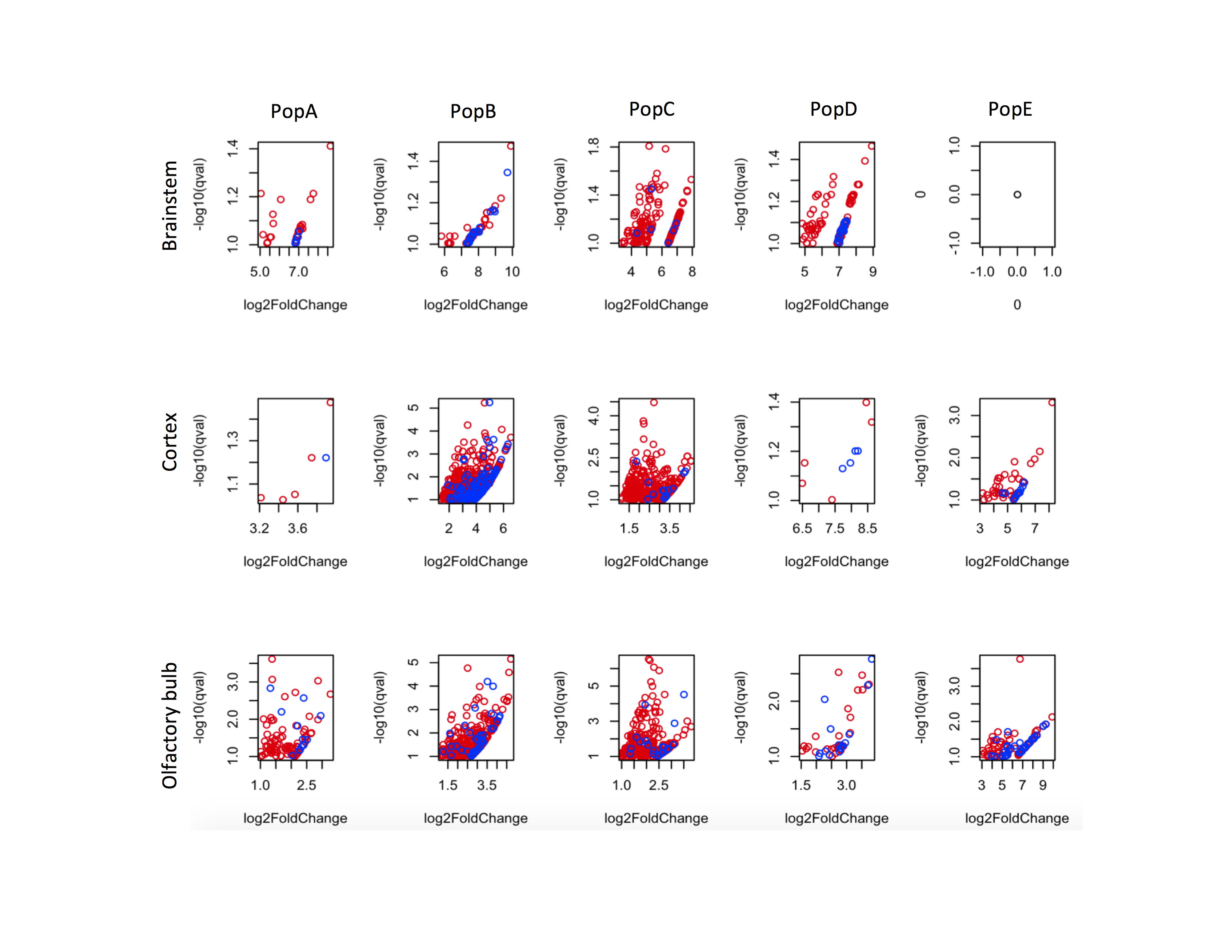

Supplement: Figure 2-2 — Comparison of log-transformed fold-changes and q values between DE lncRNA and protein-coding genes from different astrocyte subpopulation and region gene signatures. Blue circles represent DE lncRNAs and red circles represent DE protein-coding genes. Using an unpaired two-tailed t test we observed that the fold-changes of DE lncRNAs (blue) are statistically higher (p < 0.05) than those of protein-coding genes (red). Download Figure 2-2, TIF file. [file sup_enu-eN-NWR-0288-18-s02.tif]

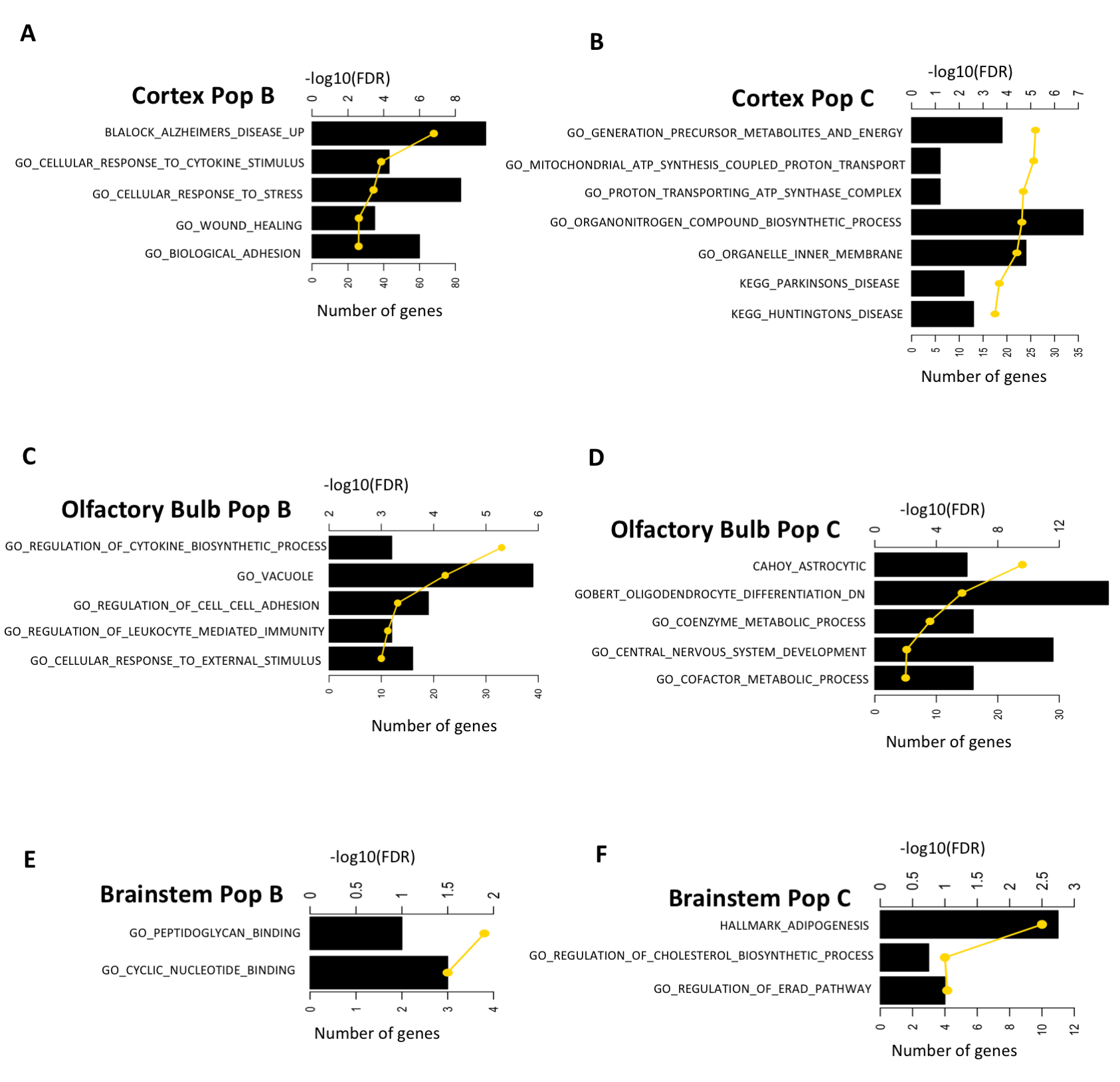

Supplement: Figure 2-3 — Gene set enrichment using astrocyte subpopulation regional gene signatures. Bar plots indicate the number of genes found in the enriched gene set. The yellow line illustrates the gene set enrichment using -log10-transformed FDR. Download Figure 2-3, TIF file. [file sup_enu-eN-NWR-0288-18-s03.tif]

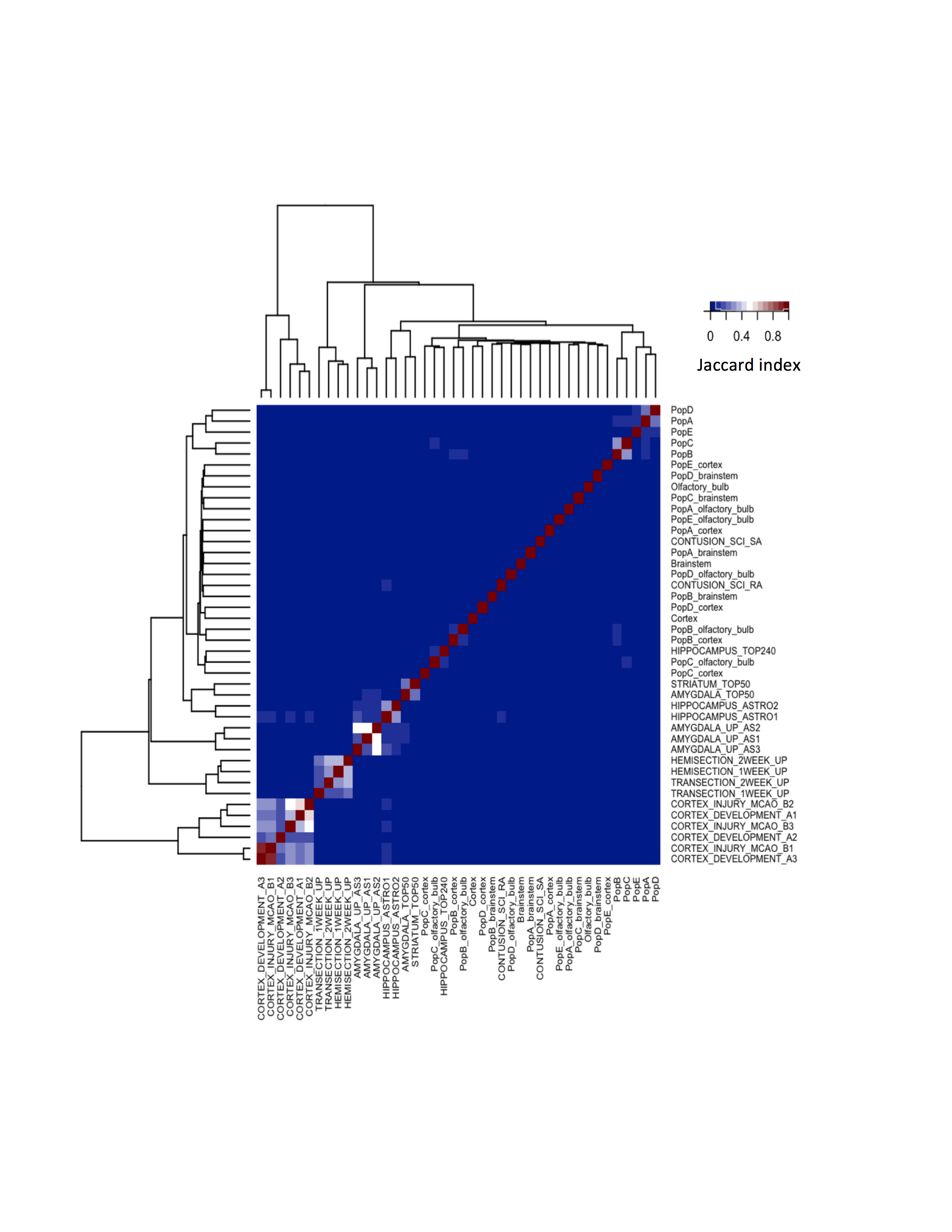

Supplement: Figure 3-2 — Heatmap displaying the Jaccard index of genes shared between astrocyte gene signatures. Download Figure 3-2, TIF file. [file sup_enu-eN-NWR-0288-18-s06.tif]

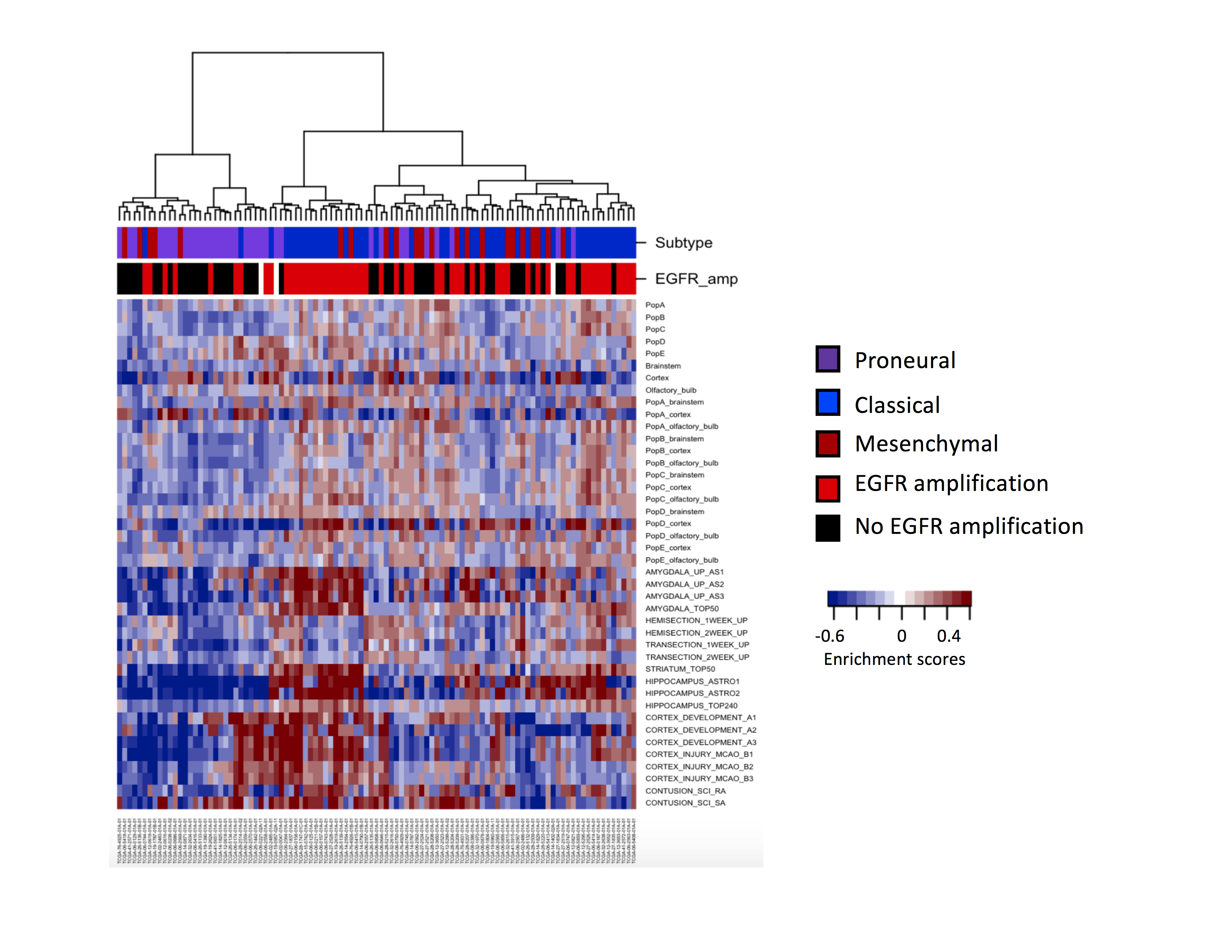

Supplement: Figure 3-3 — Heatmap displaying the hierarchical clustering of enrichment scores obtained through GSVA and GBM samples. High scores indicate strong positive correlation between astrocyte gene signatures and gene expression profiles of 103 TCGA GBM samples with a tumor purity >70%. Top bars indicate tumor subtype and the presence or absence of an EGFR gene amplification. Download Figure 3-3, TIF file. [file sup_enu-eN-NWR-0288-18-s07.tif]

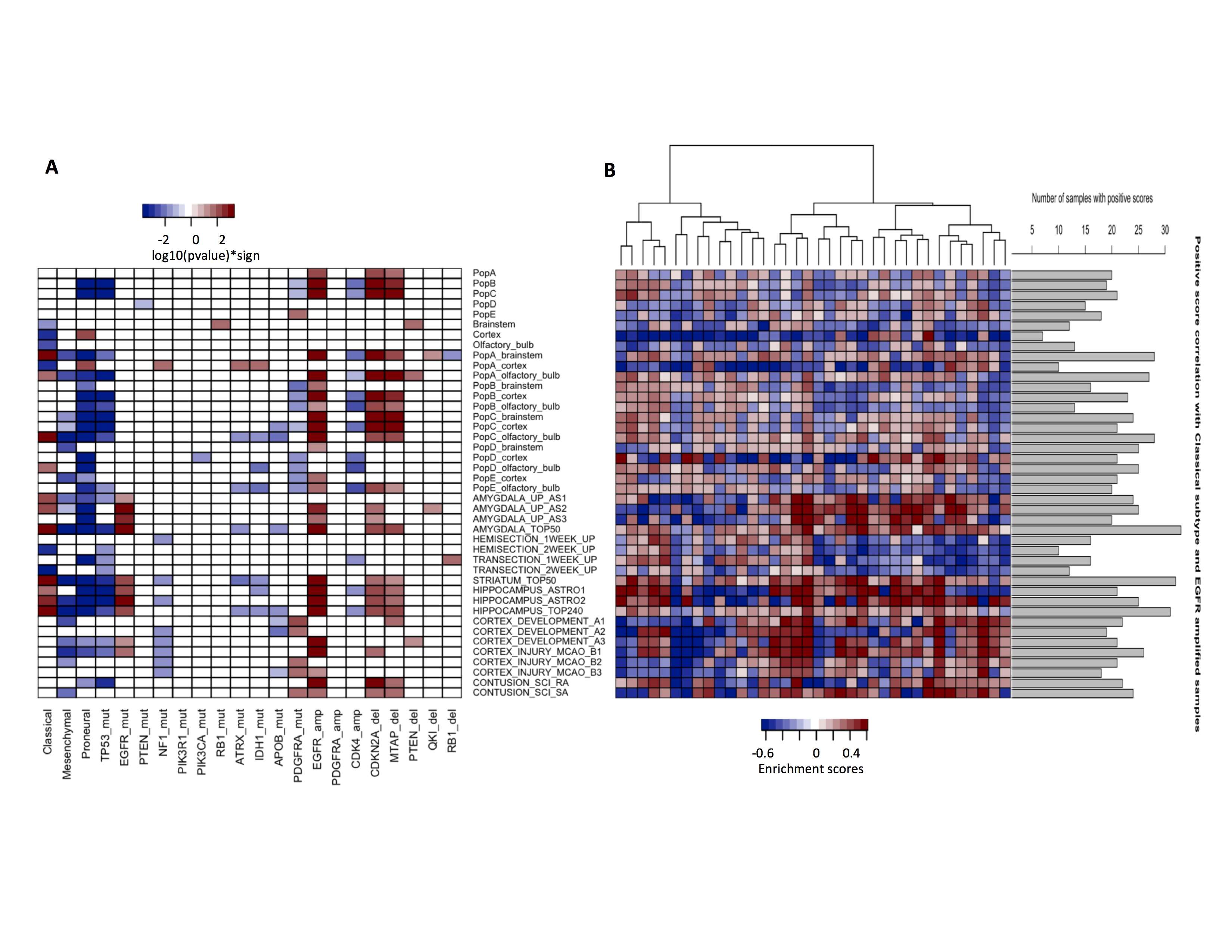

Supplement: Figure 3-4 — Correlation between upregulated astrocyte gene signatures and TCGA GBM samples. A, Analysis of the correlation between astrocyte gene signatures and GBM samples with different subtypes, somatic mutations, and copy-number variations. The heatmap depicts the -log10-transformed p value of either an ANOVA comparison between GBM subtypes or a Wilcoxon rank sum test between samples carrying the selected mutations, amplifications, and deletions. B, Heatmap representing the enrichment scores between astrocyte gene signatures and Classical GBM samples that carry an EGFR gene amplification. Bar plots indicate the number of samples with positive enrichment score for each astrocyte gene signature. Download Figure 3-4, TIF file. [file sup_enu-eN-NWR-0288-18-s08.tif]

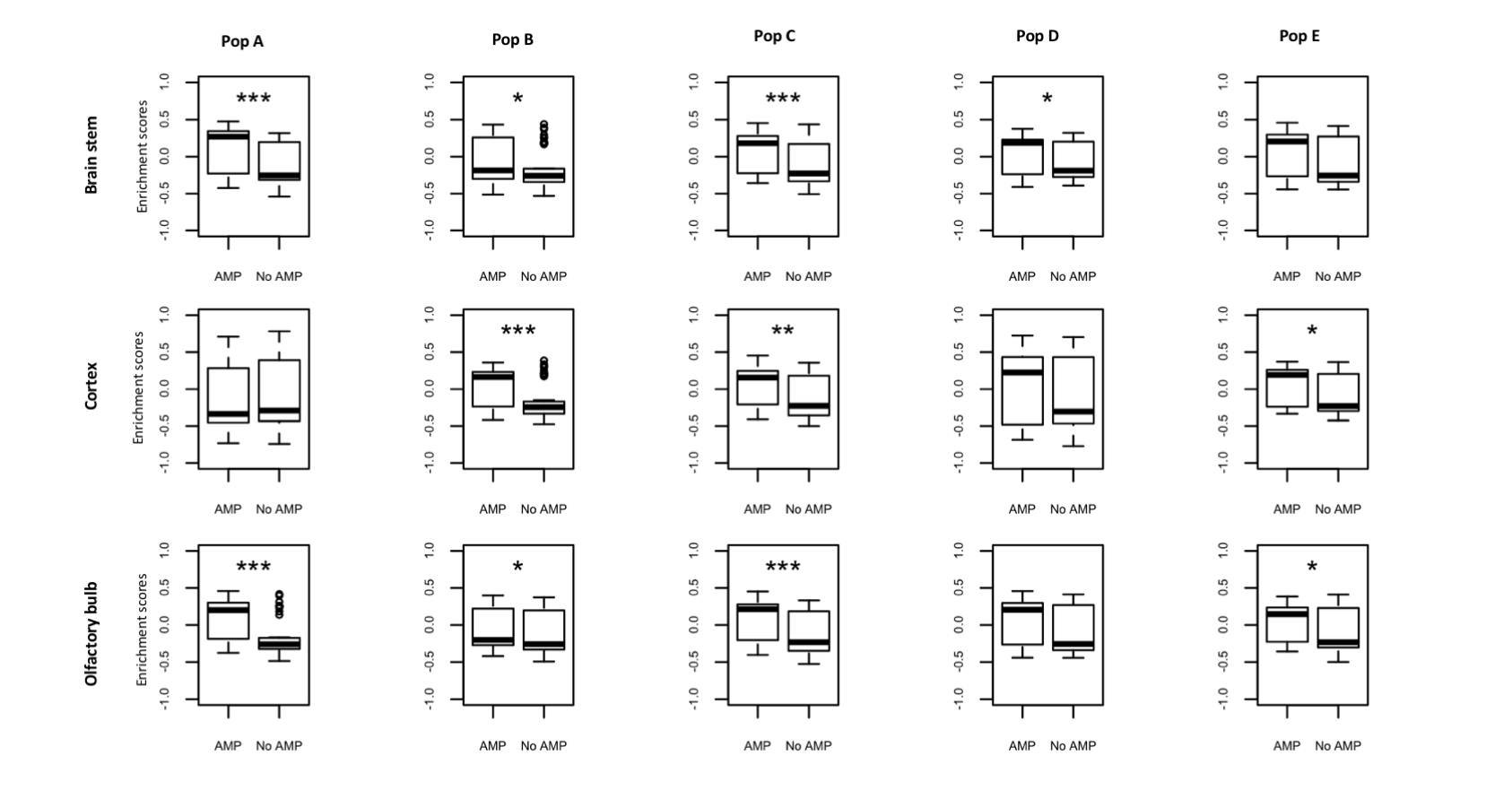

Supplement: Figure 3-5 — Correlation of region-specific astrocyte subpopulation enrichment scores with 103 TCGA Glioblastoma samples with tumor purity >70%. A Wilcoxon rank sum test was used to compare the distribution of enrichment scores between samples with and without amplification of EGFR. AMP, Amplification of EGFR; No AMP, no amplification of EGFR found. ***p < 0.001, **p < 0.01, *p < 0.05. Download Figure 3-5, TIF file. [file sup_enu-eN-NWR-0288-18-s09.tif]

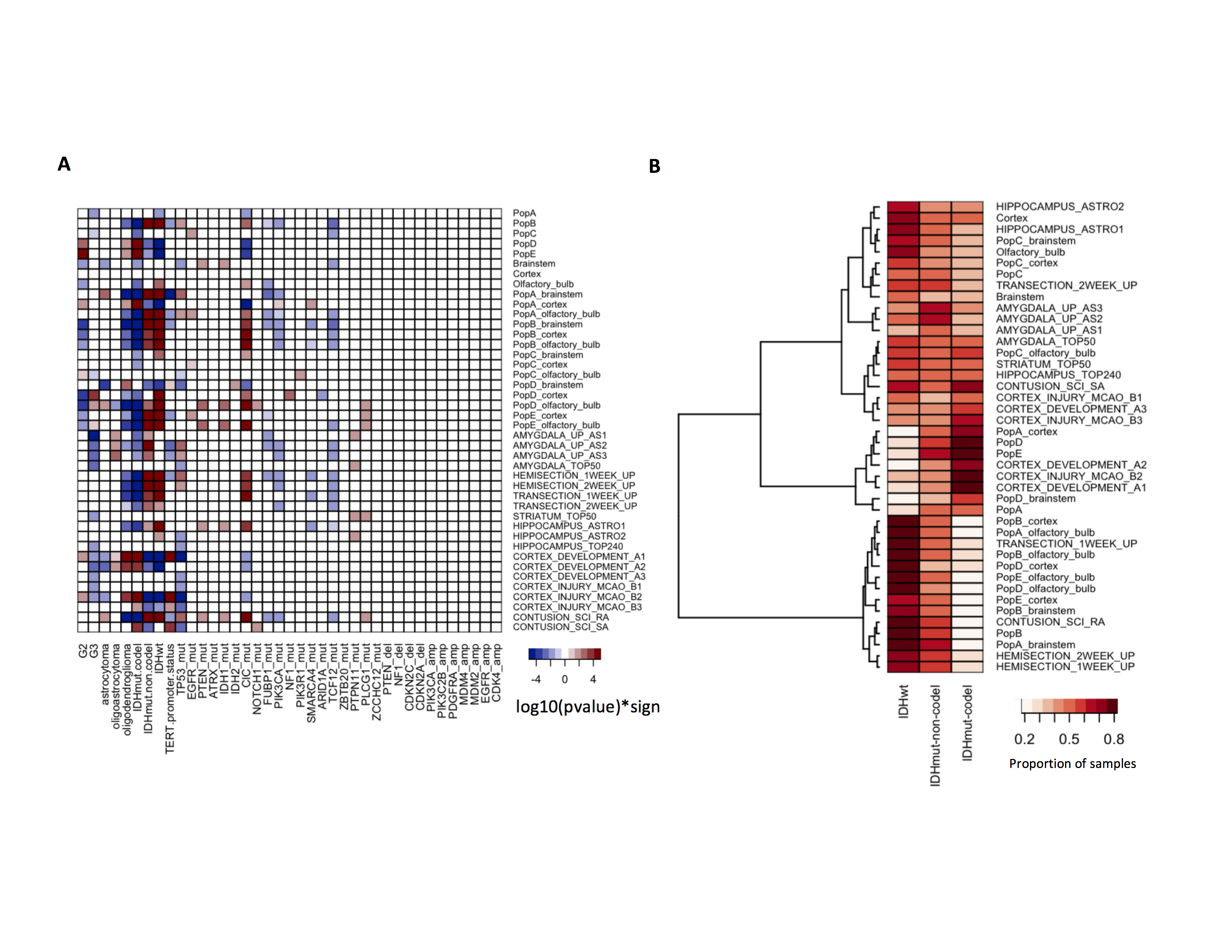

Supplement: Figure 4-1 — Correlation between upregulated astrocyte gene signatures and TCGA LGG samples. A, Analysis of the correlation between astrocyte gene signatures (y-axis) and LGG grades, histological subtypes, somatic mutations, and copy-number variations (x-axis). The heatmap depicts the -log10-transformed p value of either an ANOVA comparison between LGG subtypes or a Wilcoxon rank sum test between samples carrying the selected mutations, amplifications, and deletions. B, Heatmap depicting the proportion of LGG samples with specific IDH status variants that had positive enrichment scores for every astrocyte gene signature. Download Figure 4-1, TIF file. [file sup_enu-eN-NWR-0288-18-s11.tif]

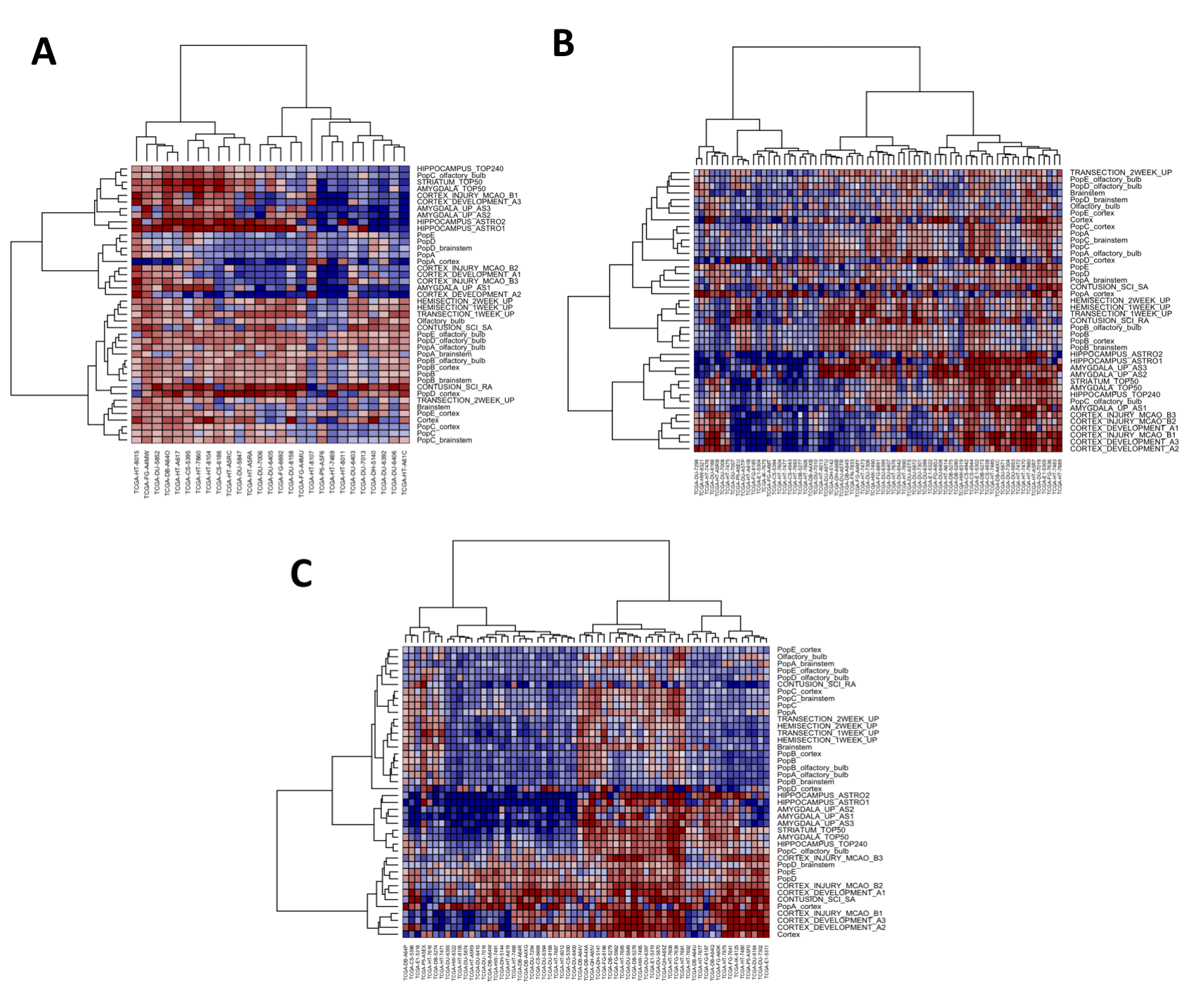

Supplement: Figure 4-2 — Heatmaps depicting GSVA enrichment score matrices between astrocyte gene signatures and TCGA LGG samples. Samples with (A) wild-type IDH1 (B) mutant IDH1 with 1p/19q codeletion, and (C) mutant IDH1 without 1p/19q codeletion are compared. Download Figure 4-2, TIF file. [file sup_enu-eN-NWR-0288-18-s12.tif]

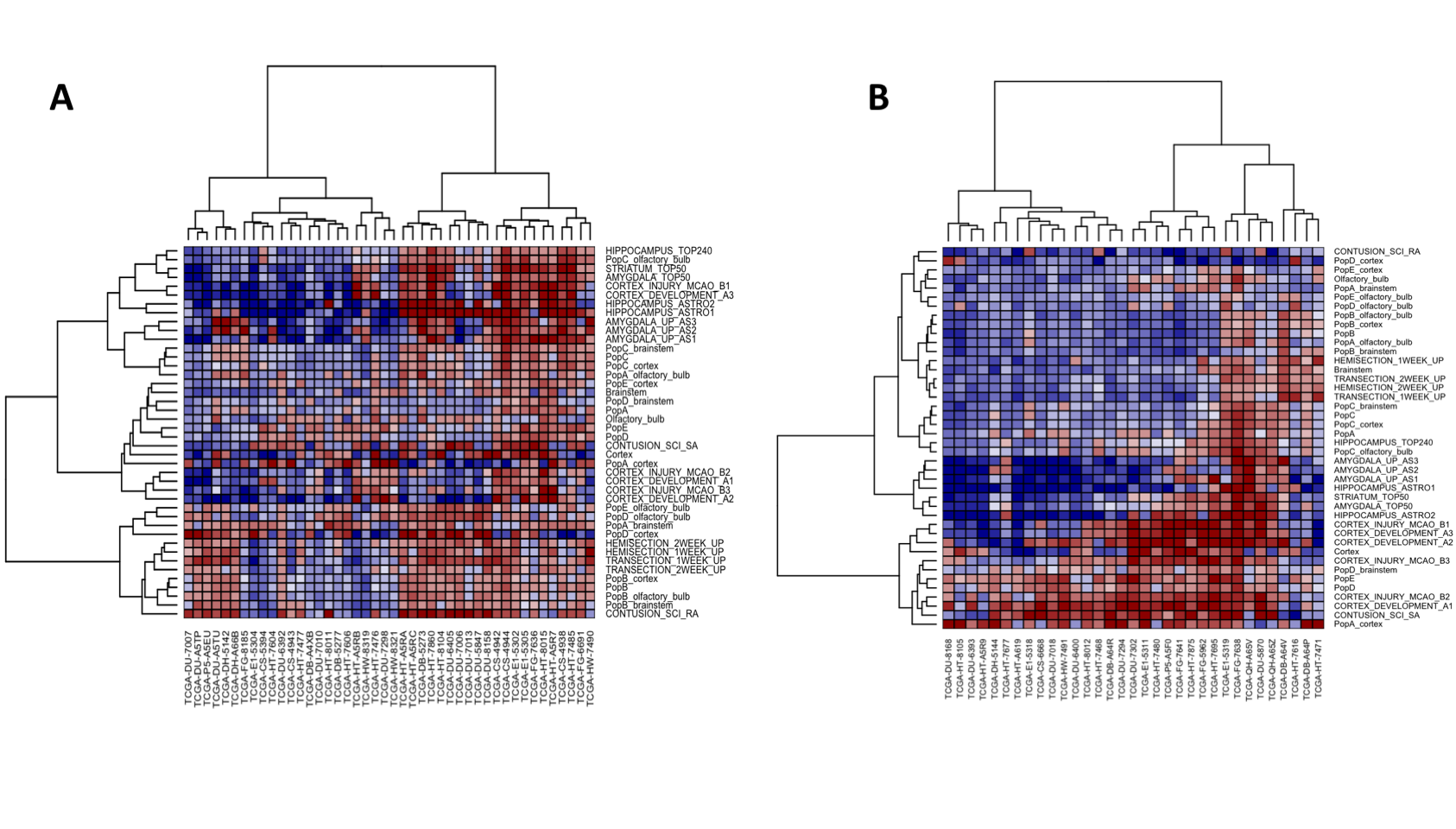

Supplement: Figure 4-3 — Heatmaps depicting GSVA enrichment score matrices between astrocyte gene signatures and TCGA LGG samples with different features. Samples with (A) astrocytoma histology and wild-type IDH1or mutant IDH1 without 1p/19q codeletion, and (B) oligodendroglioma histology and mutant IDH1 with 1p/19q codeletion, mutant TERT promoter, and mutations in CIC gene are compared. Download Figure 4-3, TIF file. [file sup_enu-eN-NWR-0288-18-s13.tif]

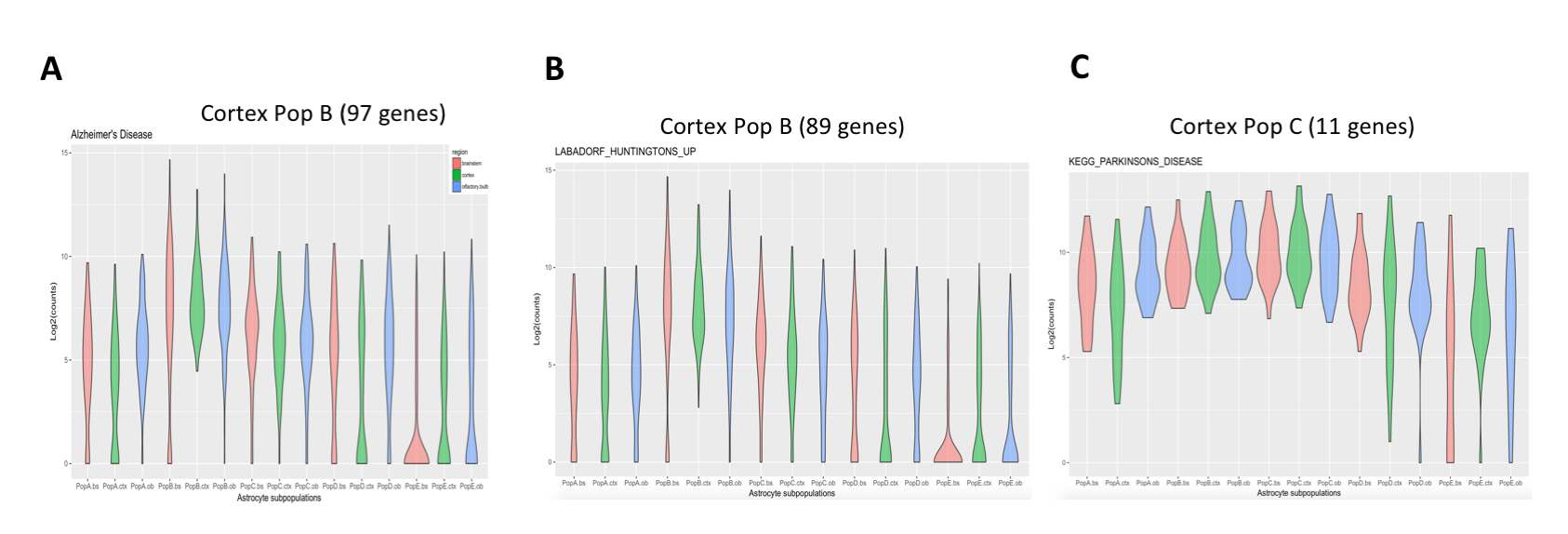

Supplement: Figure 6-1 — Violin plots representing normalized count distributions. Normalized count distributions of (A) the 97 upregulated genes from the cortex subpopulation B gene signature found in the Alzheimer’s disease gene set (B) the 87 upregulated genes from the cortex subpopulation B gene signature found in the Huntington’s disease gene set, and (C) the 11 upregulated genes from the cortex subpopulation C gene signature found in the Parkinson’s disease gene set are shown in violin plots. Download Figure 6-1, TIF file. [file sup_enu-eN-NWR-0288-18-s14.tif]

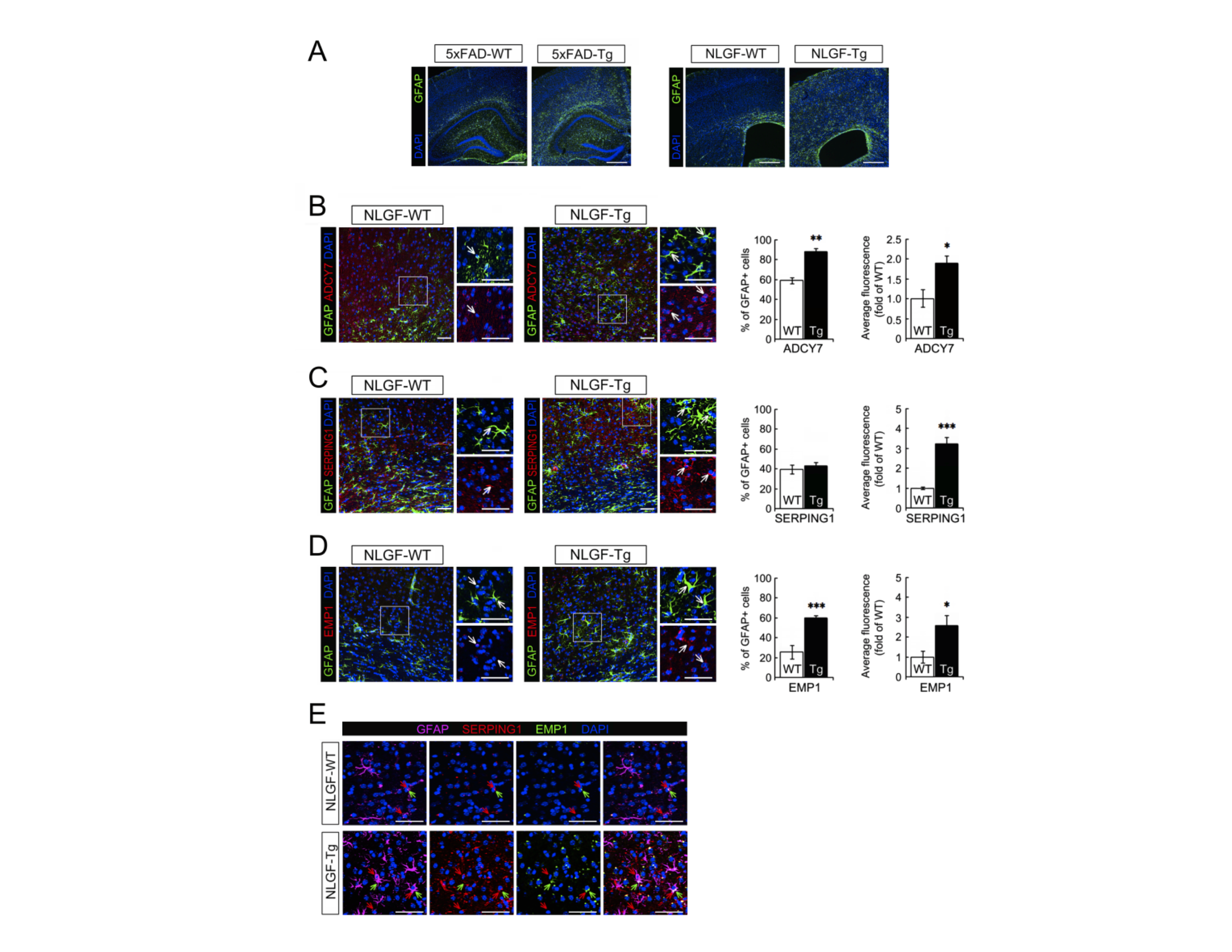

Supplement: Figure 6-2 — Protein expression of candidate genes in the brain tissues from the NLGF Alzheimer’s disease mouse model. A, AD pathology was confirmed in the cortex as an increase in GFAP+ astrocytes as well as detectable hypertrophy in 5xFAD and NLGF AD mouse models. Brain tissues collected from NLGF mouse models were prepared for immunofluorescence using anti-GFAP (green) and either (B) anti-Adcy7 (C) anti-Serping1, or (D) anti-Emp1. The regions outlined with a square are displayed at higher-magnification on the right, and the arrows point to the same cells for comparison in the fluorescent images. For the astrocytes in the inner layer of cortex (the area near the corpus callosum), the proportion of GFAP+ cells displaying red fluorescence was calculated, and the intensity of red fluorescence overlapping with GFAP signals was measured. Two-tailed unpaired Student’s t tests were used to compare AD samples to the wild-type group. E, Coimmunostaining of cortical brain tissues was performed with anti-GFAP, anti-Serping1, and anti-Emp1. *p < 0.05, **p < 0.01, ***p < 0.001. Scale bar (in A–D), 50 μm. Download Figure 6-2, TIF file. [file sup_enu-eN-NWR-0288-18-s15.tif]
